# Comparison of clinical geneticist and computer visual attention in assessing genetic conditions

**DOI:** 10.1371/journal.pgen.1011168

**Published:** 2024-02-27

**Authors:** Dat Duong, Anna Rose Johny, Suzanna Ledgister Hanchard, Christopher Fortney, Kendall Flaharty, Fabio Hellmann, Ping Hu, Behnam Javanmardi, Shahida Moosa, Tanviben Patel, Susan Persky, Ömer Sümer, Cedrik Tekendo-Ngongang, Hellen Lesmann, Tzung-Chien Hsieh, Rebekah L. Waikel, Elisabeth André, Peter Krawitz, Benjamin D. Solomon

**Affiliations:** 1 Medical Genetics Branch, National Human Genome Research Institute, Bethesda, Maryland, United States of America; 2 Institute for Genomic Statistics and Bioinformatics, University Hospital Bonn, Rheinische Friedrich-Wilhelms-Universität Bonn, Bonn, Germany; 3 Social and Behavioral Research Branch, National Human Genome Research Institute, Bethesda, Maryland, United States of America; 4 Chair for Human-Centered Artificial Intelligence, University of Augsburg, Augsburg, Germany; 5 Division of Molecular Biology and Human Genetics, Stellenbosch University, Stellenbosch, South Africa; 6 Department of Medical Genetics, Tygerberg Hospital, Tygerberg, South Africa; HudsonAlpha Institute for Biotechnology, UNITED STATES

## Abstract

Artificial intelligence (AI) for facial diagnostics is increasingly used in the genetics clinic to evaluate patients with potential genetic conditions. Current approaches focus on one type of AI called Deep Learning (DL). While DL- based facial diagnostic platforms have a high accuracy rate for many conditions, less is understood about how this technology assesses and classifies (categorizes) images, and how this compares to humans. To compare human and computer attention, we performed eye-tracking analyses of geneticist clinicians (n = 22) and non-clinicians (n = 22) who viewed images of people with 10 different genetic conditions, as well as images of unaffected individuals. We calculated the Intersection-over-Union (IoU) and Kullback–Leibler divergence (KL) to compare the visual attentions of the two participant groups, and then the clinician group against the saliency maps of our deep learning classifier. We found that human visual attention differs greatly from DL model’s saliency results. Averaging over all the test images, IoU and KL metric for the successful (accurate) clinician visual attentions versus the saliency maps were 0.15 and 11.15, respectively. Individuals also tend to have a specific pattern of image inspection, and clinicians demonstrate different visual attention patterns than non-clinicians (IoU and KL of clinicians versus non-clinicians were 0.47 and 2.73, respectively). This study shows that humans (at different levels of expertise) and a computer vision model examine images differently. Understanding these differences can improve the design and use of AI tools, and lead to more meaningful interactions between clinicians and AI technologies.

## Author summary

Artificial intelligence (AI) is increasingly used in medicine. In clinical practice, medical geneticists often use AI tools to help them examine the facial features of a patient who might have a genetic condition. While these tools are very popular, less is understood about how they work, and which parts of the face are most important to the AI tools, especially compared to medical geneticists. To address this, we performed a study where medical geneticists (as well as non-clinicians) looked at pictures of people with and without genetic conditions. We used eye-tracking tools to visualize which parts of the images the medical geneticists preferentially looked at. We compared this to the parts of the image that were the most important to the AI tools. We found that the medical geneticists and the AI tools tended to pay attention to very different features. We also found that medical geneticists and non-clinicians look at different parts of the images. Understanding how AI tools examine images compared to clinicians can help ensure the tools function properly, and could also help perform tasks like alerting medical geneticists to important clinical findings that could otherwise be overlooked.

## Introduction

Deep learning (DL), one subtype of artificial intelligence (AI), is extensively and increasingly employed in many biomedical areas. For example, generative language models show promise for analysis of medical records and related data [1]. For genomic data, DL methods been used for protein modeling and variant classification [2,3]. In medical genetics, clinicians and researchers often employ DL-based approaches to generate differential diagnoses based on facial images of individuals with possible genetic conditions [4,5].

Despite controversies and unsettled questions, healthcare is poised for major near-term changes as AI is implemented into workflows [1]. One crucial issue involves understanding how AI tools perform and how “choices” made by AI compare with those made by humans, including people with different levels of expertise [6–8]. For example, generative language models may sometimes present coherent but incorrect or inconsistent information [9]. As another example, an analysis of DL to assess chest X-rays for signs of COVID-19 showed that classifiers can be highly accurate compared to humans but may rely heavily on confounding information, such as radiographic clues outside of the lung fields [10]. Interestingly, though DL analysis of facial images has been a major application in medical genetics, relatively little work has been done to compare how clinicians view images compared to DL models [11].

To further explore this topic, we used an eye-tracking device to measure human visual attention when inspecting images of individuals with and without genetic conditions. Clinicians in our research group first selected images of several relatively common and recognizable genetic conditions, which were then viewed by other genetic medical experts as well as non-clinicians. We further trained a DL classifier to predict the ground-truth labels of these images and computed the corresponding saliency map for each image. Saliency maps allow the visualization of which parts of an image are important to the model when “deciding” how to classify an image.

First, we examined how well the visual attention maps of genetic medical experts (termed “clinicians” here) align with the classifier saliency maps. Second, we compared the visual activity of clinicians and non-clinicians. The comparison metrics for the visual maps are Intersection-over-Union (IoU) and Kullback–Leibler divergence (KL). IoU binarizes the visual maps and measures the amount of overlap between two heat maps, whereas KL treats the heat maps as continuous probability distributions and calculates the divergence between these two distributions. For both metrics, we found that saliency maps differ substantially from regions of human attention and that clinicians and non-clinicians assess images differently.

## Results

### Data collection

In this paper, we selected 10 images for the eye-tracking experiments, one for each of the following conditions: 22q11.2 deletion syndrome (22q11DS, OMIM #188400), Beckwith-Wiedemann syndrome (BWS, OMIM #130650), Cornelia de Lange syndrome (CdLS, OMIM #122470), Down Syndrome (DS, OMIM #190685), Kabuki syndrome (KS, OMIM #147920), Noonan syndrome (NS, OMIM #163950), Prader-Willi syndrome (PWS, OMIM #176270), Rubinstein-Taybi syndrome (RSTS1, OMIM #180849), Wolf-Hirschhorn syndrome (WHS, OMIM #194190), and Williams syndrome (WS, OMIM #194050). Next, six additional images of unaffected individuals selected to represent images of similarly aged individuals with similar photographic poses were also chosen as the control group (see more details in Materials and Methods and references for image sources in **S1 Text**). In summary, there are 16 images to be tested in our eye-tracking experiment.

### Human participant performance

We divided the human participants into two main groups: 22 clinicians (15 from NIH and seven from Bonn) and 22 non-clinicians. **Table 1** compares the accuracy of the two groups at identifying whether an image shows a person affected or unaffected with a genetic condition. Overall, the clinician group was more accurate than the non-clinician group at recognizing whether an image showed an affected person (correct identification was 85.6% for clinicians versus 76.9% for non-clinicians, p = 0.0032 by Chi-square). In further responding to a question about what specific genetic condition the image might depict, non-clinicians were, unsurprisingly, usually unable to identify the disease names. However, some non-clinicians are familiar with specific conditions based on their work, such as involvement in genetic research.

**Table 1:** Summary of results for each image viewed. For a specific image, we count the number of clinicians and non-clinicians who correctly and incorrectly identified that this image is of an affected or unaffected individual. Column *Identified condition name* indicates the number of clinicians and non-clinicians who correctly identified the specific condition. As eye-tracking results for seven individual images had to be discarded for technical reasons, not every image has a complete data from 22 clinicians and 22 non-clinicians. The images are listed in the order shown to the participants.

	Clinicians			Non-clinicians		
	Correct	Incorrect	Identified condition name	Correct	Incorrect	Identified condition name
**WS**	17	5	4	13	9	3
**Unaffected**	22			19	3	
**RSTS1**	21		3	21	1	
**Unaffected**	19	3		14	8	
**WHS**	20	2		19	3	
**Unaffected**	22			20	2	
**CdLS**	22		10	22		5
**DS**	22		18	21	1	1
**Unaffected**	15	6		15	6	
**KS**	19	3	11	13	9	2
**NS**	21		9	21		
**Unaffected**	18	4		18	4	
**22q11DS**	21	1	3	19	3	
**PWS**	16	5		10	12	
**Unaffected**	14	8		19	3	
**BWS**	8	13	1	5	17	

**abbreviations**: BWS: Beckwith-Wiedemann syndrome; CdLS: Cornelia de Lange syndrome; DS: Down Syndrome; KS: Kabuki syndrome; NS: Noonan syndrome; PWS: Prader-Willi syndrome; RSTS1: Rubinstein-Taybi syndrome; WHS: Wolf-Hirschhorn syndrome; WS: Williams syndrome

For the rest of the analysis, we divide all the participants into four subgroups: clinicians and non-clinicians who correctly or incorrectly identified that an image represents a person with a genetic condition. We use the terms *successful* and *underperforming clinicians* and *non-clinicians* to refer to these subgroups, respectively. For two different test images, the same participant may fail to recognize the disorders in just one of these images. Hence, for two different images, the groups of successful clinicians (and likewise non-clinicians) may not have the same participants.

**Fig 1:**
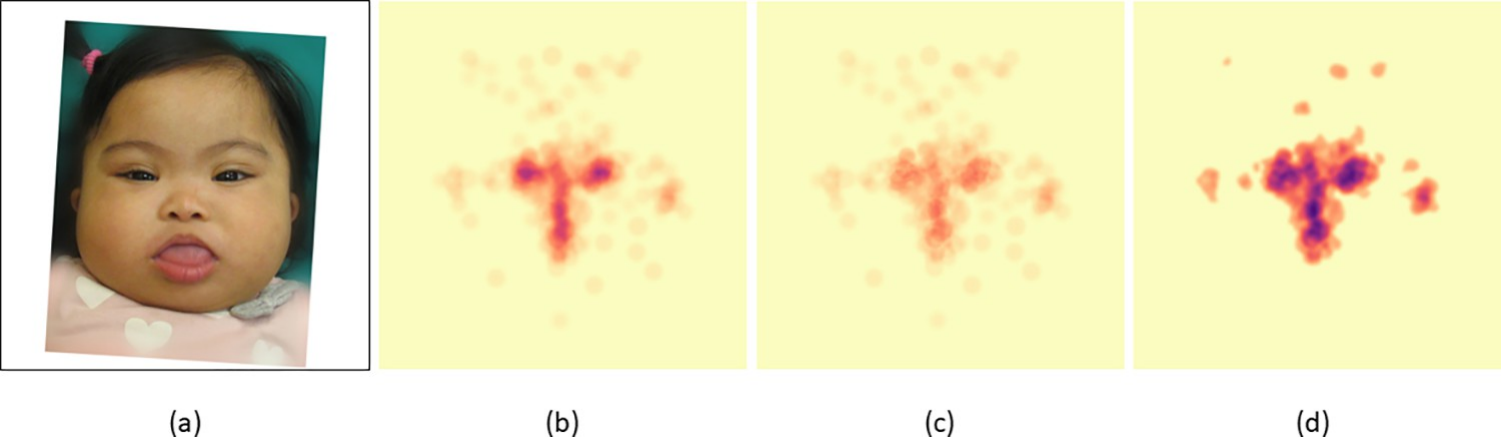
Example image illustrating the preprocessing step of visual heat maps for subsequent analyses. (a) Original image of an individual with Down syndrome (DS). (b) For this DS image, conditioned on the group of successful clinicians, we average the default attention heat maps from the eye-tracking experiments. (c) We removed the common visual signals (See **S27 Fig**). (d) Finally, we smooth the image with cv2.boxFilter and increase the color intensity. Here, compared to (b), in (d) we can better observe the high visual interest for a larger orbital region (not just the center of the eyes), as well as attention to the ears, which, though barely visible in this image, can be notable features on physical examination for this condition. The image is freely available for reuse without restriction, courtesy: National Human Genome Research Institute (www.genome.gov).

**Fig 2:**
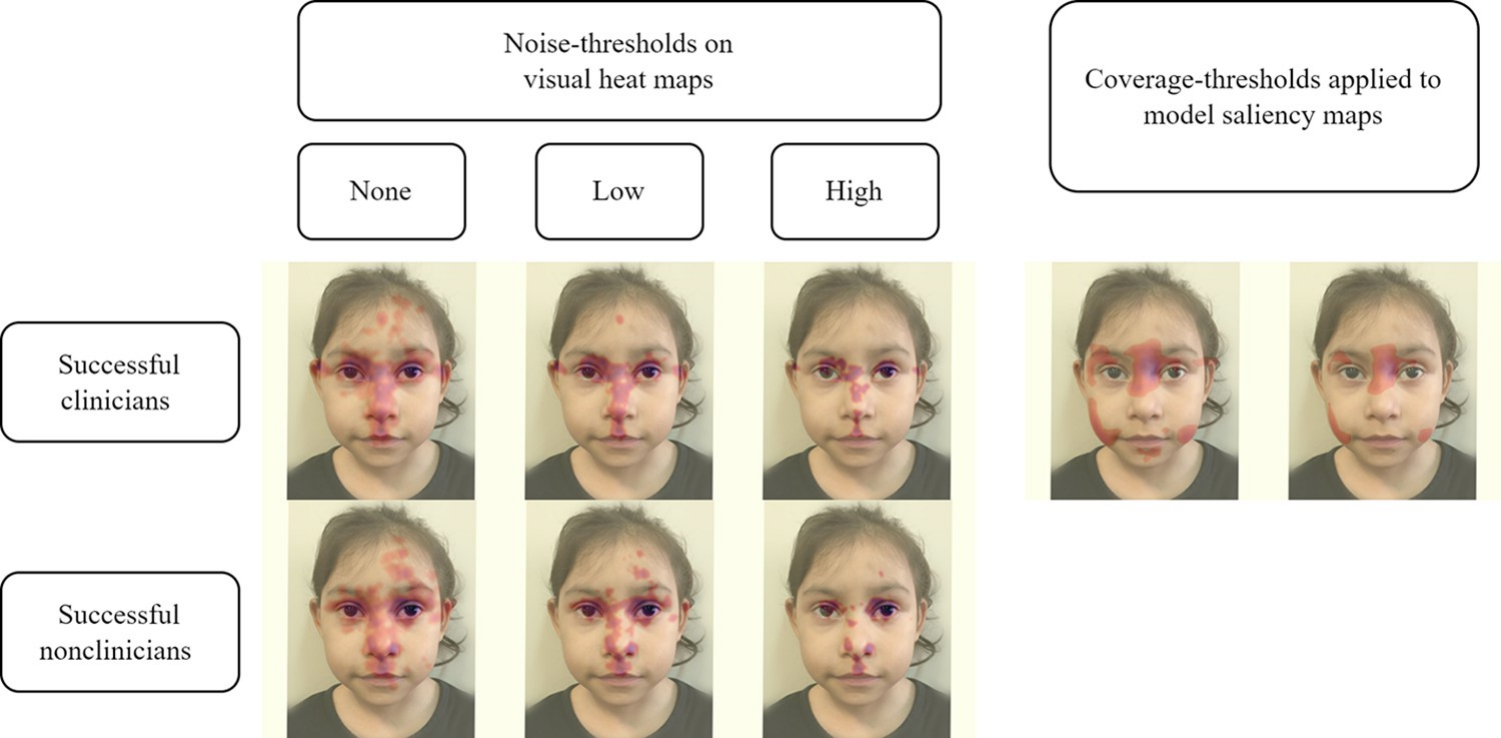
Application of noise-thresholds to human heat maps and coverage-thresholds to model saliency maps. Eye gaze heat maps are shown for the 22q11DS image used in our experiment. The left-most column shows the original average heat maps for the successful clinicians and non-clinicians. Low noise-threshold removes possibly spurious visual interests, and then the higher noise-threshold better indicates facial regions with the most prominent visual attentions. Two coverage-thresholds were applied to the model saliency map of this image; the objective is to match the number of positive pixels in the saliency maps with respect to the clinician visual attentions at the low and high-noise thresholds, respectively. Pixels below the coverage-threshold were set to zero, and the remaining number of positive pixels are approximately equal to that of the corresponding human visual map. The image is freely available for reuse without restriction, courtesy: National Human Genome Research Institute (www.genome.gov).

To understand the average visual behavior of a specific participant group (e.g., successful clinician group), we computed the average heat map for each test image over all the participants within the group (see **Fig 1** and the Materials and Methods section for further details about our data preprocessing approach). Two noise-threshold levels were applied, where a pixel value below the threshold is set to 0. The lower noise-threshold removes spurious visual signals. The higher noise-threshold removes a large proportion of the signal; thus, we would analyze only facial regions with the highest visual attention (**Figs 2** and **S1–S10**).

### Deep learning classifier performance

Since datasets and/or source codes of widely clinically used DL approaches in medical genetics cannot be easily shared (e.g., for the tool available via Face2Gene, https://www.face2gene.com/), we trained our own image classifier in this paper. Our model was trained to predict (identify) 11 different labels (10 diseases and one unaffected group). When considering unaffected and affected as the only two label choices, our classifier achieves 100% accuracy. Related to the model’s identification of specific conditions, our classifier correctly labeled 13 out of the 16 test images with the specific genetic condition that person has; averaging over these 13 images, the prediction probability of the ground truth label is 0.7853. The three misclassified affected images are CdLS, PWS, and RSTS1. For these three images, the predicted probabilities of the ground truth label and incorrect label were: CdLS (0.3196 vs 0.3285 misclassified as NS), PWS (0.3383 vs 0.4690 misclassified as WS), and RSTS1 (0.0005 vs 0.7976 misclassified as NS). Because the classification accuracy of RSTS1 is low, we excluded this image in the saliency map analysis in the next section (**S11 Fig** contains the analysis for all the affected images). See more details in the Materials and Methods section below and **S1 Text**).

### Evaluating human visual attention versus saliency map

For a specific input image, one can extract the corresponding saliency map from an image classifier (see details in the Materials and Methods section, including under the *Classifier and saliency maps* heading). This saliency map indicates the regions of the image that the model deems to be relevant for the prediction of the ground truth label. Our goal is to observe (1) which regions of an image affect the prediction probability of the ground-truth label and (2) how well these regions align with the areas most viewed by human clinicians. Hence, the saliency maps were made with respect to the ground-truth labels, whether or not the model correctly identifies these ground-truths.

For the analyses, we treat the saliency maps as if they were heat maps. We note the following key observation. Human visual heat maps produced via the Tobii eye-tracking software are partially binarized; that is, most of the visual map is blank (i.e., zero pixel value), with only certain key areas showing a color gradient (i.e., positive pixel value) to indicate key visual activity. We applied the occlusion saliency method to compute the positive contribution of every patch of pixels of an image toward the prediction of its ground-truth label. Hence, an occlusion saliency map typically contains a larger number of positive pixels than the human visual heat map. Highly unequal numbers of positive pixels between two inputs can lead to unreliable IoU and/or KL metrics. Hence, we applied a coverage-threshold to the saliency map, where pixel value below the coverage-threshold is set to 0 and the remaining number of nonzero pixels is equal to that of the human visual map **(Fig 2)**.

**Fig 3:**
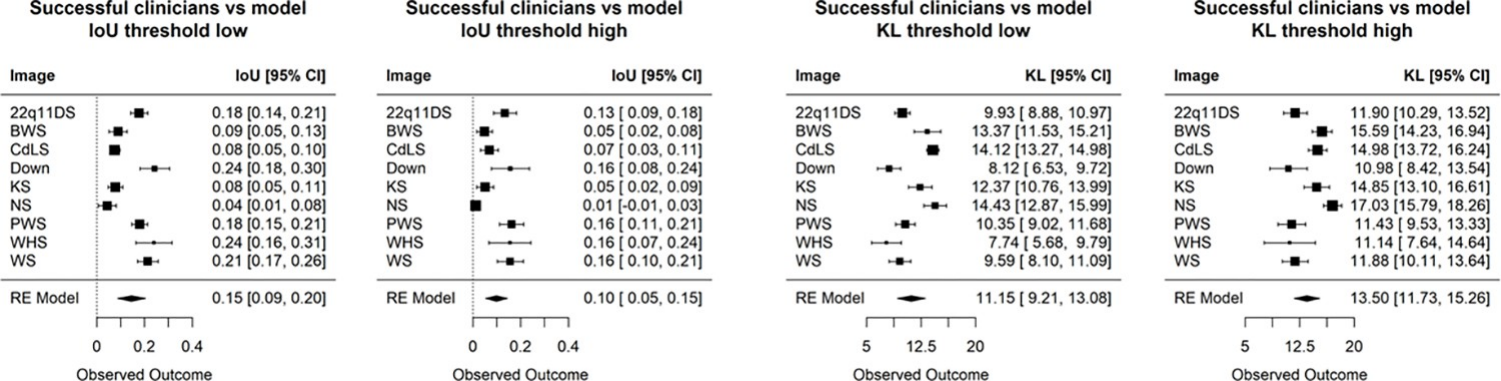
IoU and KL metric comparing the visual attention of successful clinicians and model saliency maps over 9 test disease. A low and high noise-threshold were applied to the clinician heat maps, removing spurious and retaining only highest visual activity, respectively (see **Fig 2**). At each noise-threshold of a particular human visual map, an independent coverage-threshold was applied to the corresponding model saliency map. This coverage-threshold ensures that a saliency map has equal number of positive pixels as its corresponding human visual map. At both noise-thresholds, IoU and KL are close to and far from zero, respectively, indicating that quantitatively there are differences between human and model heat maps.

For a specific test image of an affected individual, we computed the IoU score and KL divergence between the saliency map and the average heat map of the successful clinician group at both low and high noise-thresholds. At each noise-threshold of the human visual attention, we used grid-search to find the corresponding coverage-threshold for the saliency map, so that the number of nonzero pixels in the saliency map is approximately equal to that of the human visual attention. To compute IoU, pixel values above zero were converted into 1; and to compute KL, the pixel values were rescaled into a proper probability distribution. More details are explained in the Materials and Methods, with results shown in **Fig 3** (see also **S12 Fig**).

**Fig 3** shows the IoU and KL metrics averaged over the 9 affected images (excluding RSTS1 for reason mentioned above). We observed significant differences between human visual attention and what a DL classifier considers important. For example, the average IoU metric between successful clinicians versus the saliency map were 0.15 and 0.10 at low and high noise-threshold, respectively. This IoU metric is much less than the metric comparing our clinicians and non-clinician groups (see below). In other words, human heat maps show that our participants tended to look at very different regions than the classifier per the models we examined. The KL metric also indicates the same trend, where we observe a high KL value, indicating a large difference between human visual interest and model saliency maps.

### Comparing groups of human participants

We also compared the successful clinicians and non-clinicians for each of the 16 test images, and then summarized the visual attention differences over all these images. Here, we do not apply a coverage-threshold because heat maps of two participant groups may naturally have very different coverages. For example, it is entirely possible that, compared to the experts, non-clinicians’ visual activities are more dispersed since they may not recognize the signs of the genetic conditions. In **Figs 2 and S1–S10**, qualitatively speaking, the visual attentions of these clinicians and non-clinicians are more similar at a lower noise-threshold, where only spurious visual signals were removed, and later become more different at a higher noise-threshold, where only regions with high visual interests were retained. (Note that figures in **S1–S10** and **S13–S23 Figs** are shown as segmented versions of the original images due to journal policies regarding resharing of publicly available images; sources of the original images are provided in the **S1 Text** file. Segmentation for the visualization was performed via previously published methods. [12]). Thus, as shown in **Figs 2 and S1–S10,** although these clinicians and non-clinicians correctly recognize affected versus unaffected individuals, visually speaking, on average these two participant groups do not appear to display the same trend when assessing the images.

**Fig 4:**
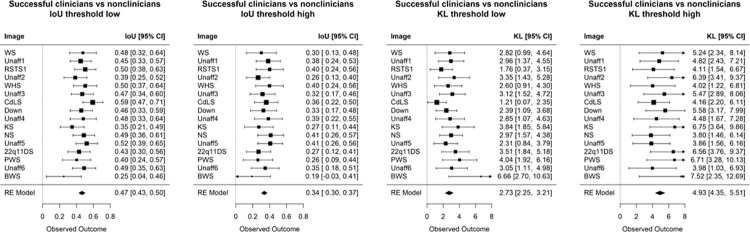
Comparison of visual heat maps of successful (correct) human participants at different noise-threshold levels. IoU and KL metric compare the visual heat maps of successful clinicians versus successful non-clinicians. When removing only low spurious signal (“threshold low”), the two groups display more similar visual interests. However, when considering only regions with high visual interests (“threshold high”), the two groups differ more drastically (average IoU drops from 0.47 to 0.34, and average KL increases from 2.73 to 4.93). The images are listed in the order in which images were shown to the participants.

**Fig 4** numerically compares the visual interests of successful clinicians versus successful non-clinicians. Here, the result aligns with our qualitative inspection in **Fig 2**. When excluding low spurious signals with a low noise-threshold, these two subgroups show similar eye-gaze interests (IoU = 0.47, and KL = 2.73). However, these two subgroups become more different when considering only the highest visual attention (IoU dropped to 0.34, and KL increased to 4.93). A similar trend is observed when we analyzed on just the set of affected and unaffected images (**S12 Fig**).

**Fig 5:**
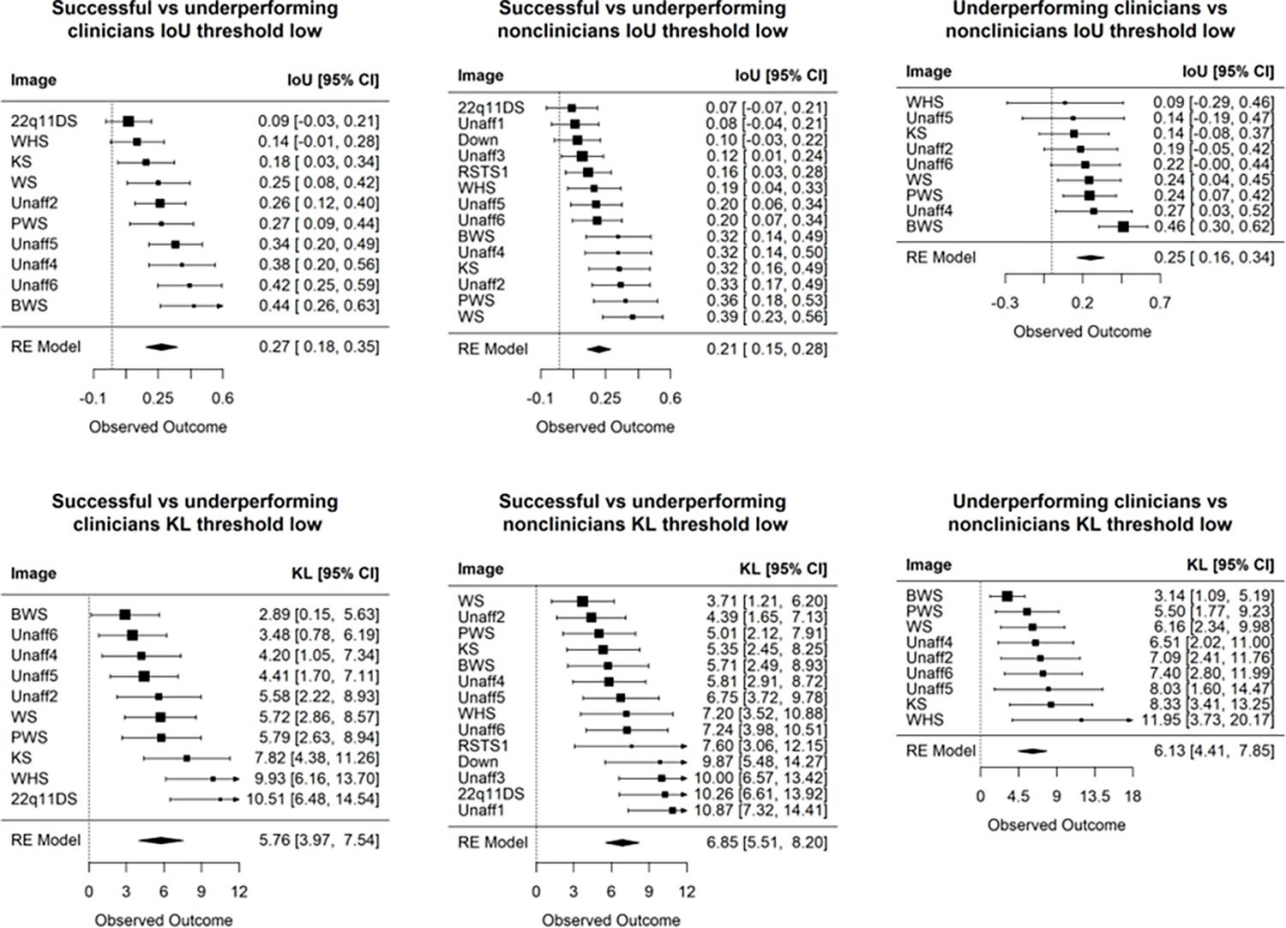
Additional comparisons of human participants. Images are sorted based on the IoU (top row) and KL (bottom row), which compare the heat maps between different groups of participants. A low noise-threshold was applied to remove spurious visual activity from the average heat map of each group. These three comparisons show lower similarity scores than that of successful clinicians versus successful non-clinicians (IoU = 0.47, and KL = 2.73 in **Fig 4**). Clinicians tend to show more similar visual interests for WS, PWS, BWS, and unaffected images (left column). This trend is not seen for non-clinicians (middle column).

We further compare the successful and underperforming clinician group. **Fig 5** shows that, on average, these two subgroups of clinicians do not have similar visual interests. The same trend is seen when stratifying the non-clinicians according to their accuracy. Conditioned only on participants who misclassified the images, **Fig 5** also shows visual attention differences between clinicians and non-clinicians. Hence, when participants misclassify images, they have distinct ways of inspecting the images that do not appear to be similarly influenced by some common confounders in the images (e.g., hairstyles, facial expressions, or earrings that could affect a participant’s decision). Overall, there seems to be more similar visual attention when a participant (whether a clinician or not) can correctly identify that a person is affected by a genetic condition; for example, the IoU values in **Fig 4** are higher than those in **Fig 5**.

We also examined aggregated heat maps produced by each of the clinicians and non-clinicians and evaluated whether there were different visual patterns when the same participant examined affected or unaffected images. As shown in **Figs S24–S26** show that participants had different patterns (ways) of assessing images.

Finally, we examined the AOIs that corresponded to specific dysmorphic features in the images of affected individuals (**S14–S23 Figs**). As each test image has more than one AOI that may be important to the underlying conditions, metrics like duration-of-fixation and time-to-first-whole-fixation for any single AOI can have high standard deviations, making it difficult to observe statistical significance. Overall, we did not detect a specific pattern for these AOIs that differentiated the categories of participants.

## Discussion

In clinically oriented analyses, the performance of DL and other AI models have traditionally been compared to that of human experts, such as evaluating how well DL models versus humans recognize the presence of metastatic cancer from lymph node sections or assess chest X-rays from inpatient and urgent care settings [13,14]. There has overall been less attention to comparing the *processes* of computers and humans when performing tasks. One reason for this involves the fact that, when DL classifiers emerged, they were not originally designed, nor were there available saliency approaches, to enable easy visual explanation of their processes. More recently, saliency methods were developed independently of the DL classifiers, and have been explored in a variety of biomedical contexts, including related to multiple types of radiologic studies (e.g., computed tomography, magnetic resonance imaging, retinal imaging, and X-rays), electrocardiograms, and in a variety of pathology analyses [15]. These studies primarily focused on comparing saliency results to the portions of biomedical images that had been annotated to contain the most clinically relevant data, such as specific cell types in pathology images, but paid less attention to the overall process [16]. This overall process may be important to consider to investigate questions like pertinent positives and negatives, as well as to search for potential confounders.

Against this background, our study has two key results. First, human and computer attention differs substantially when evaluating images of individuals with potential genetic conditions. This does not mean that one is superior to the other, but these types of analyses and metrics may be helpful in future studies. For example, methods to compare human and computer attention can be used to explore potential confounders in AI-based analyses, or to develop methods that improve the accuracy or applicability of AI tools. As AI is increasingly adopted in clinical scenarios, such studies will be critical to assess model performance. For generalizability, such future studies would need to be much larger, both in terms of the number of participants and the number of images and genetic conditions included.

Second, clinicians and non-clinicians exhibit different gaze behaviors when assessing images. This is not surprising, but quantifying these behaviors using methods like these may be helpful for activities such as ascertaining which phenotypic characteristics may be diagnostically important but which are frequently overlooked. Again, as AI support enters more and more clinical areas, these types of analyses may point to specific ways to augment the relationship between clinicians and AI tools. For example, data from extremely high-performing clinicians in human/classifier comparison experiments may be useful to design the education of less experienced clinicians or trainees, as well as AI tools [17].

This study has several limitations. These include the number of participants and images viewed. The images also represent heterogeneous genetic conditions, and eye-tracking behavior may be affected by certain aspects of a particular image. While we grouped clinicians and non-clinicians into separate categories, there is varying experience and expertise within these groups, and differences in behavior between individuals. Additionally, our analyses focused on metrics like human visual attention, which may not reflect what is most important to a person evaluating an image. For example, an expert clinician may immediately perceive a key visual clue to a diagnosis and may then move on to spend more time observing less obvious features or searching the image for subtle clues.

From the DL perspective, we do not claim that our model is highly accurate or represents the best possible option–our aim was to compare what a DL model weights when analyzing images to what a human looks at, and we intentionally chose images with a range of performance on our DL classifier, as well as images that we subjectively felt would have a range of difficulty for human participants. Along these lines, saliency map analyses were not performed with respect to the correct and incorrect predicted label, separately. Rather, the DL saliency map analyses were performed with respect to the ground-truth labels, whether or not the ground-truths are the model’s correct prediction. The only requirement was that our model’s predicted probabilities for these ground-truths needed to be larger than zero. Otherwise, the saliency map may not be reliable when the model fails to make any prediction for this ground-truth label [15]. In this current study, the chosen images each have a set of key condition-specific features unique to that specific condition, and each condition is relatively distinct from each other. In future studies, we plan to select a larger number of conditions that have more shared syndromic features among several different conditions (e.g., many different conditions that all involve facial findings like hypertelorism or upslanted palpebral fissures), which would let us better understand and compare the behavior of the model and humans, such as when they both incorrectly classify an image. Overall, while our results show interesting trends, due to small number of test images, caution should be taken that they will generalize to other or all genetic conditions, to larger groups of individuals, or to other DL models or AI-based approaches.

As mentioned above, the results here point to multiple future possibilities, including involving larger numbers of participants and datasets to analyze further how well the results can be extrapolated. Related eye-tracking experiments could be used to explore multiple questions germane to genetics, such as with different data types (e.g., radiological studies or other physical examination features) encountered in clinical practice. Additional work could be done specifically quantifying different classifiers and saliency methods.

## Materials and methods

### Ethics statement

This study was approved as IRB exempt by the IRBs of the National Institutes of Health (NIH) (Bethesda, Maryland, United States) and the University of Bonn (Bonn, Germany). NIH study ID: 000846; Bonn study ID: 210/23-EP. All participants were adults (the images viewed were of previously published pediatric individuals); formal consent was not required, with subjects agreeing to participate by taking part in the study.

### Data collection

Similar to our previous methods [18,19], we selected publicly available images of individuals between 2 and 18 years of age affected by one of 10 genetic conditions (for reference, we provide the first OMIM #ID for conditions with multiple OMIM # IDs): 22q11.2 deletion syndrome (22q11DS, OMIM #188400), Beckwith-Wiedemann syndrome (BWS, OMIM #130650), Cornelia de Lange syndrome (CdLS, OMIM #122470), Down Syndrome (DS, OMIM #190685), Kabuki syndrome (KS, OMIM #147920), Noonan syndrome (NS, OMIM #163950), Prader-Willi syndrome (PWS, OMIM #176270), Rubinstein-Taybi syndrome (RSTS1, OMIM #180849), Wolf-Hirschhorn syndrome (WHS, OMIM #194190), and Williams syndrome (WS, OMIM #194050), as well as images of unaffected individuals similar in age and ancestral diversity to the affected individuals. The genetic conditions were chosen as they represent relatively common genetic conditions that involve recognizable craniofacial features, and with which geneticists would be expected to be at least somewhat familiar [20]. Two geneticists (BDS, CTN) and a genetic counselor (RLW) on the study team selected the images to represent typical images representing the chosen conditions; images of some conditions were felt to be more difficult to recognize than others. Sixteen images (one image for each of the above conditions, and six images of unaffected individuals) were selected for the eye-tracking experiments. The selected images were at least 720-pixel resolution; they were each standardly cropped, centered, and aligned (e.g., the eyes, nose, and mouth were roughly repositioned at the same coordinates for all images See **S1 Text** for image sources; due to journal image sharing policies, images in **S1–S10 and S13–S23 Figs** are shown as segmented versions of the images used in the eye-tracking experiments [12].

### Eye tracking experiments

The formatted images were embedded in a screen-based eye-tracking system (Tobii Pro X3-120, Tobii Lab Pro version 1.194.41215; https://www.tobii.com/, Stockholm Sweden). This system uses infrared light to detect pupillary movement, which allows the analysis of gaze activity, such as how long they look at different parts of each image. Eye-tracking experiments took place in two locations: the National Institutes of Health (NIH) (Bethesda, Maryland, United States) and the University of Bonn (Bonn, Germany).

After calibration, each participant viewed the 16 images for seven seconds per image, and answered questions about each image, including whether the image showed a person affected by a genetic condition, and, if so, what condition the person had. After extensive initial testing, we chose seven seconds for the viewing time, as subjective feedback and preliminary assessments showed that this amount of time was sufficient to assess an image but minimized visually revisiting areas of the image in a way that might not further inform the assessment. To minimize head movement or distractions, questions were asked verbally during the eye-tracking portions of the experiment. Responses were documented manually by a study team member.

The NIH cohort included 17 individuals, including physician geneticists (n = 14) and physicians in genetics subspecialty training (n = 3). The Bonn cohort included 29 total individuals, including physician geneticists (n = 2) and physicians in genetics subspecialty training (n = 5), as well as 22 non-clinicians. Some of the non-clinicians in our cohort have some experience through their work in recognizing genetic conditions. For example, some are graduate students who study applications of AI in genetics, and thus are familiar with genetic conditions, but are not trained clinicians.

### Data extraction and analysis

We extracted eye-tracking data for analysis in two main ways. First, we extracted individual heat maps for each participant and image (these heat maps show the relative amount of time each participant looked at different parts of each image). We used the default Tobii software settings, but with two changes to enable our analyses: a) we changed the output eye-tracking radius to 25 pixels, as our preliminary analyses showed that the default 50 pixel radius was insufficiently precise for comparisons; b) we used a more homogeneous color scheme for the heat maps (with the following hexadecimal color codes: high: #943126, medium: #B03A2E, low: #CB4335), which enabled our quantitative analyses to take into account all captured heat map data, including to compare eye-tracking data with classifier saliency maps.

Second, to enable additional analyses that took into account gaze trajectory behavior to analyze the timing of participant gaze [21], a dysmorphologist (BDS) manually drew areas-of-interest (AOIs) for each image (see **S14–S23 Figs**). The AOI set included only those features that were listed as having dysmorphic manifestations based on the clinical synopses in OMIM (https://www.omim.org/) and which were also present in the images. For example, if a condition were listed in the clinical synopsis section of OMIM as having a dysmorphic manifestation affecting the eyebrows and that manifestation was present in the image of a person with that condition used in the survey, an AOI was drawn around the eyebrows. Using the Tobii software, we extracted tabular data for analysis based on these defined AOIs.

Prior to analysis, we manually reviewed results and excluded heat maps where eye-tracking data was not recorded (this occurred for all results for one NIH participant, which may have been due to ophthalmologic issues like severe myopia, and seven total other isolated eye-tracking results for unclear reasons).

We observed that standard heat maps primarily showed that the areas around the eyes, nose, and mouth are the regions with the highest attention. These common visual attentions (**S27 Fig**), which likely reflect standard human behavior, make it difficult to quantitatively compare the visual activity differences between groups unless accounted for. To mitigate this issue, we computed the average heat map for all the clinicians and non-clinicians (separately) over all of the images. We then subtracted this common average gaze pattern (separately for clinicians and non-clinicians) from each heat map. This does not cause us to ignore these areas of common facial attention in our analyses but helps account for typical human behavior when viewing faces. **Fig 1** explains our data preprocessing approach.

In our analyses of the images, we focused on four subgroups: clinicians and non-clinicians who correctly or incorrectly identified that an image represents a person with a genetic condition. One interest is whether the untrained intuition of a non-clinician aligns with clinician behavior. In the later sections of our analyses, we use the terms *successful* and *underperforming clinicians* and *non-clinicians* to refer to the clinicians and non-clinicians who correctly and incorrectly recognize the presence of a genetic disease for a given test image, respectively. For two different test images, the same participant may fail to recognize the disorders in both images. Hence, for two different images, the groups of successful clinicians (and likewise non-clinicians) may not have the same participants.

To understand the general behavior of a specific participant group (e.g., successful clinician group), after accounting for the areas of common visual attention, we computed the average heat map for each test image over all the participants. Two different thresholds were applied to remove noise from the heat maps, where pixel values below this threshold are set to 0. The first, lower noise-threshold is meant to remove spurious visual activities. The second, higher threshold removes a large proportion of the signal; here, we would analyze only facial regions with the highest visual attention. At each noise-threshold, to compare the average heat maps (of two participant groups or against the model), we applied two different metrics: Intersection-over-Union (IoU), and Kullback–Leibler divergence (KL).

Previous work applied IoU to benchmark human annotations against DL model saliency maps for chest X-ray images [15]. IoU is symmetric, ranges between 0 and 1, and is intuitive; that is, when similarity is high, then there is more overlap between two heat maps. We note that IoU requires the pixel values to be binarized (e.g., 0 or 1). Here, at each noise-threshold, nonzero pixels (i.e., pixels higher than the noise-threshold) are set as 1 when computing IoU. This binarization may result in the loss of intensity within both human visual heat maps and model saliency maps. Thus, we applied a second metric. Our second metric (KL) does not binarize the pixel values. Instead, KL treats the heat maps as two-dimensional probability distributions and calculates the divergence between these two distributions [22]. Unlike IoU, KL is not symmetric and ranges from 0 to infinity. We opted for the symmetric implementation of KL; that is, we take the average KL (x,y) and KL (y,x) for two inputs x and y. Suppose we want to compare the average visual attention of a clinician group against a model saliency map, then the two inputs x and y are the average human heat map and the model saliency map, respectively. A KL value far from 0 implies that human visual map and model saliency map are very different.

Standard deviation for the observed IoU (and likewise for KL divergence) was computed via bootstrapping. Finally, we applied random effects meta-analysis (we refer to this as “RE Model” in **Figs 3, 4, 5, S11, S12, and S26**) to summarize the behavioral differences over many images between the two groups of participants (e.g., successful clinicians versus successful non-clinicians).

### Classifier and saliency maps

Separate from the training and validation set, our test set includes the 16 images used in eye-tracking experiments and 2–4 additional images for several selected conditions. With the remaining images in the dataset (not containing any test images), we applied 5-fold cross validation to train 5 different models. For each iteration of the 5-fold cross validation, we finetuned EfficientNet-B4 pre-trained on ImageNet with respect to the other 4 folds and validate it on the left-out fold. EfficientNet-B4 was chosen because relatively it is one of the models requiring a relatively low number of parameters while still obtaining high accuracy on ImageNet [23]. Each image was resized to 448 x 448 pixels, and cross-entropy loss with equal weights for the diseases was used. Grid-search was performed to find a single set of hyperparameters that works best on average over all the 5-folds. We found the following hyperparameters to work best: batch size 64, dropout rate 0.2, and learning rate 0.00003 for Adam optimizer [24]. Training scripts are provided in the GitHub links (see Data and code availability section). **Table A** in **S1 Text** shows the top-k accuracy (acc@k) on the validation set.

After training 5 models from 5-fold cross validation, we built an ensemble classifier by averaging the predictions of these models for a test image. **Table B** in **S1 Text** shows the model performance for the images used in eye-tracking experiments (**Table C** in **S1 Text** shows performance on additional selected test images). Again, we aimed to compare which portions of an image were important to a DL classifier versus human eye-tracking attention, and different models would be expected to return different results; limitations for our classifier are that [1] our model misclassifies some images and [2] even when the predictions are correct, the predicted probabilities for the ground-truth labels may not be close to 1.

The occlusion saliency method with box size 20 x 20 pixels and stride 10 x 10 was used to produce the saliency maps [25]. The intuition is to remove a 20 x 20 pixel box from the image (e.g., set the pixel values within this box to 0) and then measure how much the prediction accuracy drops. This approach allows us to identify key regions affecting the classifier output. For example, if an image region is truly unimportant, then we should not see a change in the classifier output (e.g., the saliency map would have zero values for this region). Conversely, if an image region contains important syndromic features related to a certain genetic condition, then removing this region would decrease the prediction probability of this condition. In this case, the image region is considered to be positively contributing toward the prediction of the genetic condition (e.g., the saliency map would have nonzero values for this region).

We note that, our goal is to observe: (1) which regions of an image affect the prediction probability of the ground-truth label; (2) how these regions differ from those areas viewed most by human clinicians. Hence, we did not conduct the saliency analyses on the correct predictions and then on the incorrect predictions, separately. Rather, the saliency analyses were performed with respect to the ground-truth labels regardless of whether or not the model correctly identifies these ground-truths.

A saliency map for each test image with respect to its ground-truth label was taken as the average of the saliency maps from each of the models from 5-fold cross validation. As mentioned, for a specific test image, this ground-truth label may or may not be the model’s correct prediction. We only require that the model’s predicted probability of this ground-truth to not be very close to zero; otherwise, the saliency map may not be reliable when the model fails to make any prediction for the ground-truth [15]. When comparing to a human visual heat map, we applied a coverage-threshold to the saliency map. Pixel values below this coverage-threshold are set to zero; grid-search was used to find the best coverage-threshold where the remaining number of non-zero pixels is roughly equal to that of the human visual heat map. This coverage-threshold ensures that the human visual heat map and model saliency map are contributing equal numbers of “active” pixels toward the computation of the IoU and KL metrics; otherwise, these metrics can be unreliable.

We observed that, for human visual attentions at the low noise-threshold, the number of nonzero pixels takes up about 6–8% of the face region for all the test images. Hence, for each saliency map, the coverage-threshold was set to have 7% of the face region to be nonzero pixels. At a high noise-threshold for the human visual attentions, 3–4% of the face region were nonzero pixels, and thus the coverage-threshold for each saliency map was set to have 3.5% of the face region to be nonzero pixels.

We acknowledge that there are other models besides EfficientNet-B4 that have been studied in the context of genetic and other congenital diseases [4,5]. Moreover, there are other saliency approaches besides the occlusion method. This paper’s combination of EfficientNet-B4 and the occlusion method seems to return reasonable results. In future studies, we plan to evaluate other types of image classifiers and saliency approaches.

## Supporting information

S1 Fig (a) Eye gaze heat map of the participants overlayed on a segmented version of the original 22q11DS image (see **S1 Text** for original image sources). The leftmost image is the average heat map of the successful clinicians (top) and non-clinicians (bottom). We apply two different noise-thresholds: a low threshold to remove possibly spurious visual interests (middle), and then a high threshold that more clearly indicates facial regions with high visual attentions (right). (b) Saliency maps of image with key regions that affect the classifier accuracy. We apply a low (left) and high coverage-threshold (right) on the saliency maps so that the area covered is approximately the same as the low and high noise-threshold, respectively.
(TIF)

S2 Fig (a) Eye gaze heat map of the participants overlayed on a segmented version of the original BWS image (see **S1 Text** for original image sources). The leftmost image is the average heat map of the successful clinicians (top) and non-clinicians (bottom). We apply two different noise-thresholds: a low threshold to remove possibly spurious visual interests (middle), and then a high threshold that more clearly indicates facial regions with high visual attentions (right). (b) Saliency maps of image with key regions that affect the classifier accuracy. We apply a low (left) and high coverage-threshold (right) on the saliency maps so that the area covered is approximately the same as the low and high noise-threshold, respectively.
(TIF)

S3 Fig (a) Eye gaze heat map of the participants overlayed on a segmented version of the original CdLS image (see **S1 Text** for original image sources). The leftmost image is the average heat map of the successful clinicians (top) and non-clinicians (bottom). We apply two different noise-thresholds: a low threshold to remove possibly spurious visual interests (middle), and then a high threshold that more clearly indicates facial regions with high visual attentions (right). (b) Saliency maps of image with key regions that affect the classifier accuracy. We apply a low (left) and high coverage-threshold (right) on the saliency maps so that the area covered is approximately the same as the low and high noise-threshold, respectively.
(TIF)

S4 Fig (a) Eye gaze heat map of the participants overlayed on a segmented version of the original DS image (see **S1 Text** for original image sources). The leftmost image is the average heat map of the successful clinicians (top) and non-clinicians (bottom). We apply two different noise-thresholds: a low threshold to remove possibly spurious visual interests (middle), and then a high threshold that more clearly indicates facial regions with high visual attentions (right). (b) Saliency maps of image with key regions that affect the classifier accuracy. We apply a low (left) and high coverage-threshold (right) on the saliency maps so that the area covered is approximately the same as the low and high noise-threshold, respectively.
(TIF)

S5 Fig (a) Eye gaze heat map of the participants overlayed on a segmented version of the original KS image (see **S1 Text** for original image sources). The leftmost image is the average heat map of the successful clinicians (top) and non-clinicians (bottom). We apply two different noise-thresholds: a low threshold to remove possibly spurious visual interests (middle), and then a high threshold that more clearly indicates facial regions with high visual attentions (right). (b) Saliency maps of image with key regions that affect the classifier accuracy. We apply a low (left) and high coverage-threshold (right) on the saliency maps so that the area covered is approximately the same as the low and high noise-threshold, respectively.
(TIF)

S6 Fig (a) Eye gaze heat map of the participants overlayed on a segmented version of the original NS image (see **S1 Text** for original image sources). The leftmost image is the average heat map of the successful clinicians (top) and non-clinicians (bottom). We apply two different noise-thresholds: a low threshold to remove possibly spurious visual interests (middle), and then a high threshold that more clearly indicates facial regions with high visual attentions (right). (b) Saliency maps of image with key regions that affect the classifier accuracy. We apply a low (left) and high coverage-threshold (right) on the saliency maps so that the area covered is approximately the same as the low and high noise-threshold, respectively.
(TIF)

S7 Fig (a) Eye gaze heat map of the participants overlayed on a segmented version of the original PWS image (see **S1 Text** for original image sources). The leftmost image is the average heat map of the successful clinicians (top) and non-clinicians (bottom). We apply two different noise-thresholds: a low threshold to remove possibly spurious visual interests (middle), and then a high threshold that more clearly indicates facial regions with high visual attentions (right). (b) Saliency maps of image with key regions that affect the classifier accuracy. We apply a low (left) and high coverage-threshold (right) on the saliency maps so that the area covered is approximately the same as the low and high noise-threshold, respectively.
(TIF)

S8 Fig (a) Eye gaze heat map of the participants overlayed on a segmented version of the original RSTS1 image (see **S1 Text** for original image sources). The leftmost image is the average heat map of the successful clinicians (top) and non-clinicians (bottom). We apply two different noise-thresholds: a low threshold to remove possibly spurious visual interests (middle), and then a high threshold that more clearly indicates facial regions with high visual attentions (right). (b) Saliency maps of image with key regions that affect the classifier accuracy. We apply a low (left) and high coverage-threshold (right) on the saliency maps so that the area covered is approximately the same as the low and high noise-threshold, respectively.
(TIF)

S9 Fig (a) Eye gaze heat map of the participants overlayed on a segmented version of the original WHS image (see **S1 Text** for original image sources). The leftmost image is the average heat map of the successful clinicians (top) and non-clinicians (bottom). We apply two different noise-thresholds: a low threshold to remove possibly spurious visual interests (middle), and then a high threshold that more clearly indicates facial regions with high visual attentions (right). (b) Saliency maps of image with key regions that affect the classifier accuracy. We apply a low (left) and high coverage-threshold (right) on the saliency maps so that the area covered is approximately the same as the low and high noise-threshold, respectively.
(TIF)

S10 Fig (a) Eye gaze heat map of the participants overlayed on a segmented version of the original WS image (see **S1 Text** for original image sources). The leftmost image is the average heat map of the successful clinicians (top) and non-clinicians (bottom). We apply two different noise-thresholds: a low threshold to remove possibly spurious visual interests (middle), and then a high threshold that more clearly indicates facial regions with high visual attentions (right). (b) Saliency maps of image with key regions that affect the classifier accuracy. We apply a low (left) and high coverage-threshold (right) on the saliency maps so that the area covered is approximately the same as the low and high noise-threshold, respectively.
(TIF)

S11 Fig IoU and KL metric comparing the visual attention of successful clinicians and model saliency maps over all the 10 test diseases (which includes RSTS1). A low and high noise-threshold were applied to the clinician heat maps, removing spurious and retaining only highest visual activity, respectively. At each noise-threshold of a particular human visual map, an independent coverage-threshold was applied to the corresponding model saliency map. Pixels of a saliency map below this coverage-threshold were set to zero, and the remaining number of nonzero pixels would be roughly equal to that of the corresponding human visual map. Coverage-threshold ensures that a saliency map has equal number of positive pixels as its corresponding human visual map.
(TIF)

S12 Fig IoU and KL metric comparing the heat maps (at different noise-threshold) of successful clinicians versus non-clinicians conditioned on whether the test images are of affected or unaffected individuals. On average, there is no major differences between how human participants view images of affected and unaffected individuals.
(TIF)

S13 Fig Eye gaze heat map of the participants overlayed on the unaffected images. For each set, the left most image is the average heat map of the successful clinicians (top) and non-clinicians (bottom). We apply two different noise-thresholds: a low threshold to remove possibly spurious visual interests (middle), and then a higher threshold that more clearly indicates facial regions with high visual attentions (right).
(TIF)

S14 Fig We defined the AOIs specific to the HPO-annotated features for the 22q11DS image (left image). A segmented version of the original image is shown, and the AOIs drawn on the original image may not perfectly match the segmented version shown (see **S1 Text** for original image sources). Boxplots compare duration-of-fixation and time-to-first-whole-fixation.
(TIF)

S15 Fig We defined the AOIs specific to the HPO-annotated features for the BWS image (left image). A segmented version of the original image is shown, and the AOIs drawn on the original image may not perfectly match the segmented version shown (see **S1 Text** for original image sources). Boxplots compare duration-of-fixation and time-to-first-whole-fixation.
(TIF)

S16 Fig We defined the AOIs specific to the HPO-annotated features for the CdLS image (left image). A segmented version of the original image is shown, and the AOIs drawn on the original image may not perfectly match the segmented version shown (see **S1 Text** for original image sources). Boxplots compare duration-of-fixation and time-to-first-whole-fixation.
(TIF)

S17 Fig We defined the AOIs specific to the HPO-annotated features for the DS image (left image). A segmented version of the original image is shown, and the AOIs drawn on the original image may not perfectly match the segmented version shown (see **S1 Text** for original image sources). Boxplots compare duration-of-fixation and time-to-first-whole-fixation.
(TIF)

S18 Fig We defined the AOIs specific to the HPO-annotated features for the KS image (left image). A segmented version of the original image is shown, and the AOIs drawn on the original image may not perfectly match the segmented version shown (see **S1 Text** for original image sources). Boxplots compare duration-of-fixation and time-to-first-whole-fixation.
(TIF)

S19 Fig We defined the AOIs specific to the HPO-annotated features for the NS image (left image). A segmented version of the original image is shown, and the AOIs drawn on the original image may not perfectly match the segmented version shown (see **S1 Text** for original image sources). Boxplots compare duration-of-fixation and time-to-first-whole-fixation.
(TIF)

S20 Fig We defined the AOIs specific to the HPO-annotated features for the PWS image (left image). A segmented version of the original image is shown, and the AOIs drawn on the original image may not perfectly match the segmented version shown (see **S1 Text** for original image sources). Boxplots compare duration-of-fixation and time-to-first-whole-fixation.
(TIF)

S21 Fig We defined the AOIs specific to the HPO-annotated features for the RSTS1 image (left image). A segmented version of the original image is shown, and the AOIs drawn on the original image may not perfectly match the segmented version shown (see **S1 Text** for original image sources). Boxplots compare duration-of-fixation and time-to-first-whole-fixation.
(TIF)

S22 Fig We defined the AOIs specific to the HPO-annotated features for the WHS image (left image). A segmented version of the original image is shown, and the AOIs drawn on the original image may not perfectly match the segmented version shown (see **S1 Text** for original image sources). Boxplots compare duration-of-fixation and time-to-first-whole-fixation.
(TIF)

S23 Fig We defined the AOIs specific to the HPO-annotated features for the WS image (left image). A segmented version of the original image is shown, and the AOIs drawn on the original image may not perfectly match the segmented version shown (see **S1 Text** for original image sources). Boxplots compare duration-of-fixation and time-to-first-whole-fixation.
(TIF)

S24 Fig For each of the 22 clinicians, we average heat maps over all 10 affected (a) and 6 unaffected (b) images. Each participant displays unique behavior. For example, in (a) the first participant (row 1 column 1) and sixth participant (row 2 column 1) showed unequal interest at the nose area.
(TIF)

S25 Fig For each of the 22 non-clinicians, we average heat maps over all 10 affected (a) and 6 unaffected (b) images. Each participant displays unique behavior; however, visually this uniqueness is less detectable compared to **S24 Fig**.
(TIF)

S26 Fig We applied a low noise-threshold to remove spurious signals from the visual heat maps, and then estimated the differences in the visual attention between affected and unaffected images conditioned on the same participant **(see S24 and S25 Figs)**. Participants were given random IDs of gene names. IoU and KL metric show that a few participants (e.g., clinician EP300) are more consistent with their visual behavior when viewing affected and unaffected images, suggesting that these participants may be looking at similar facial features for both types of images.
(TIF)

S27 Fig We take the average of all the visual attention heat maps across all test images for the clinician (left) and non-clinician groups (right). We observed that most of the visual interests align with eyes, nose, and mouth areas. To account for normal human behavior when viewing an image, conditioned on the group expertise (clinician or non-clinician), we subtracted these common average areas from each individual heat map used for our analyses. This helps account for typical human behavior when viewing faces but does not cause us to ignore these areas of common facial attention.
(TIF)

S1 Text Contains **Tables A-C** and additional explanations about the DL model analyses and image sources.
(DOCX)
