# Supplementary figures and images for: Comparison of clinical geneticist and computer visual attention in assessing genetic conditions
Source: PLoS Genet. 2024 Feb 27;20(2):e1011168. doi: 10.1371/journal.pgen.1011168 (PMC10923488; doi:10.1371/journal.pgen.1011168)

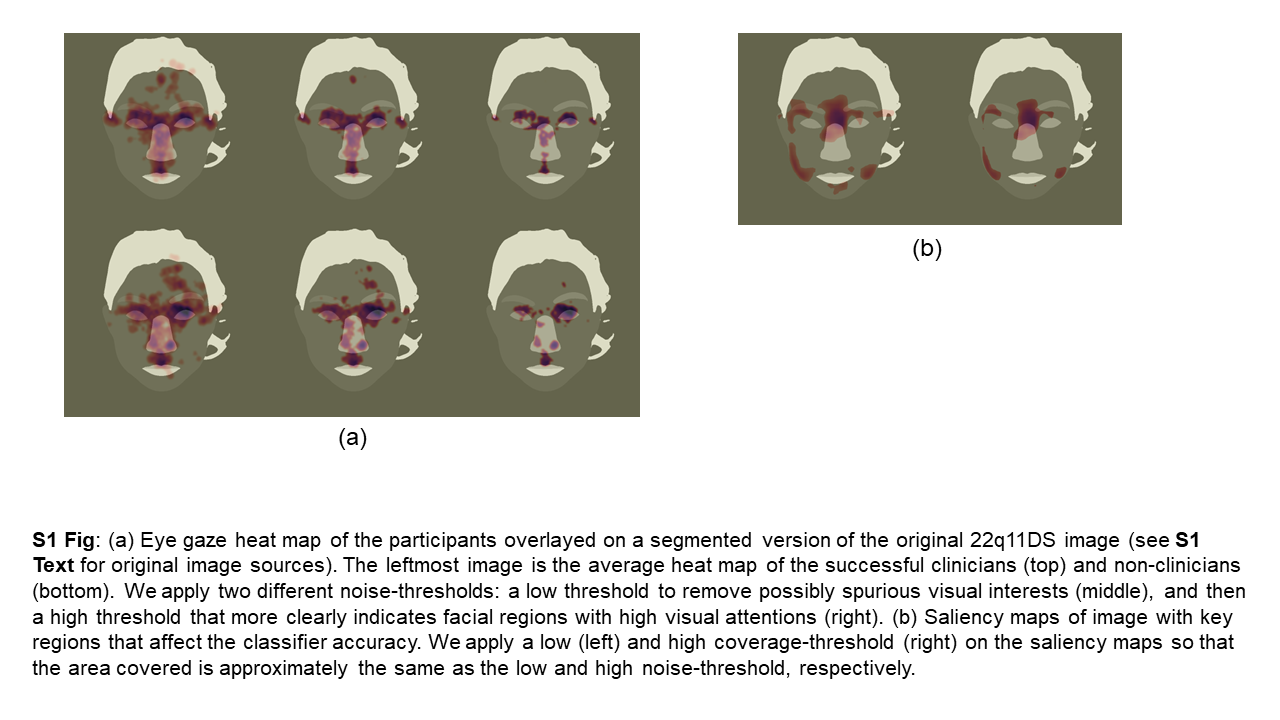

Supplement: S1 Fig — (a) Eye gaze heat map of the participants overlayed on a segmented version of the original 22q11DS image (see S1 Text for original image sources). The leftmost image is the average heat map of the successful clinicians (top) and non-clinicians (bottom). We apply two different noise-thresholds: a low threshold to remove possibly spurious visual interests (middle), and then a high threshold that more clearly indicates facial regions with high visual attentions (right). (b) Saliency maps of image with key regions that affect the classifier accuracy. We apply a low (left) and high coverage-threshold (right) on the saliency maps so that the area covered is approximately the same as the low and high noise-threshold, respectively. (TIF) [file pgen.1011168.s001.tif]

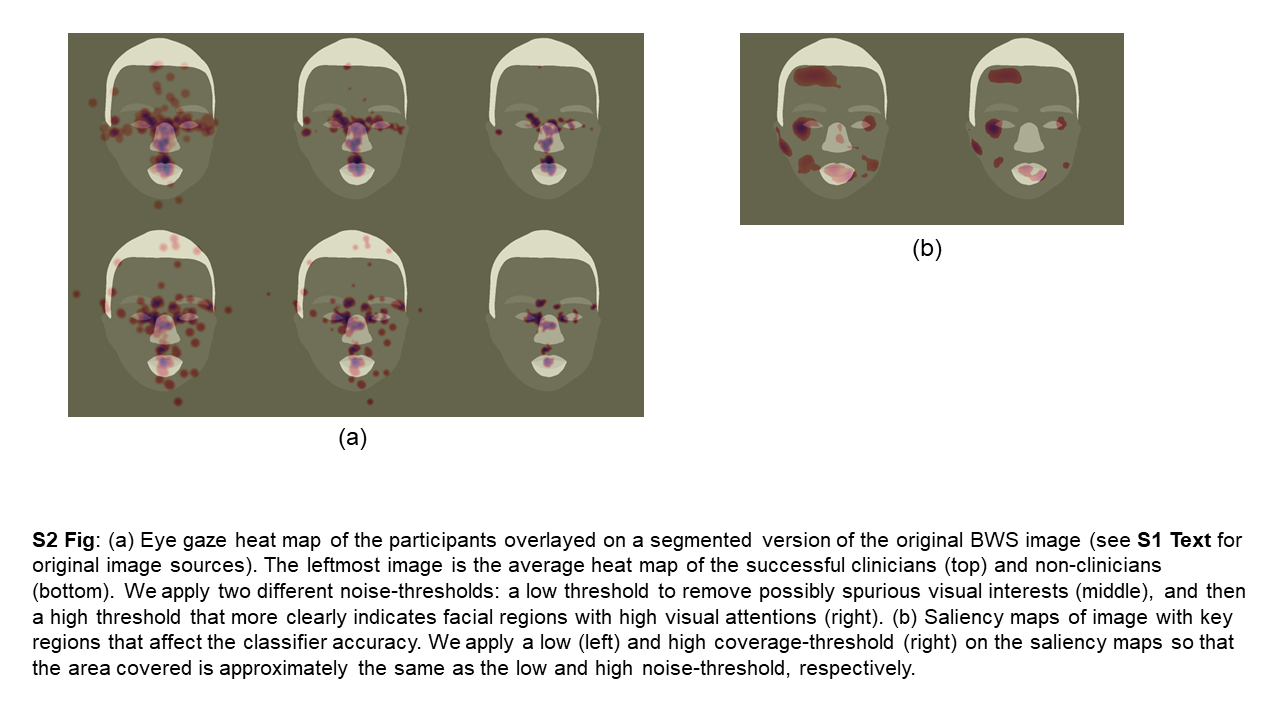

Supplement: S2 Fig — (a) Eye gaze heat map of the participants overlayed on a segmented version of the original BWS image (see S1 Text for original image sources). The leftmost image is the average heat map of the successful clinicians (top) and non-clinicians (bottom). We apply two different noise-thresholds: a low threshold to remove possibly spurious visual interests (middle), and then a high threshold that more clearly indicates facial regions with high visual attentions (right). (b) Saliency maps of image with key regions that affect the classifier accuracy. We apply a low (left) and high coverage-threshold (right) on the saliency maps so that the area covered is approximately the same as the low and high noise-threshold, respectively. (TIF) [file pgen.1011168.s002.tif]

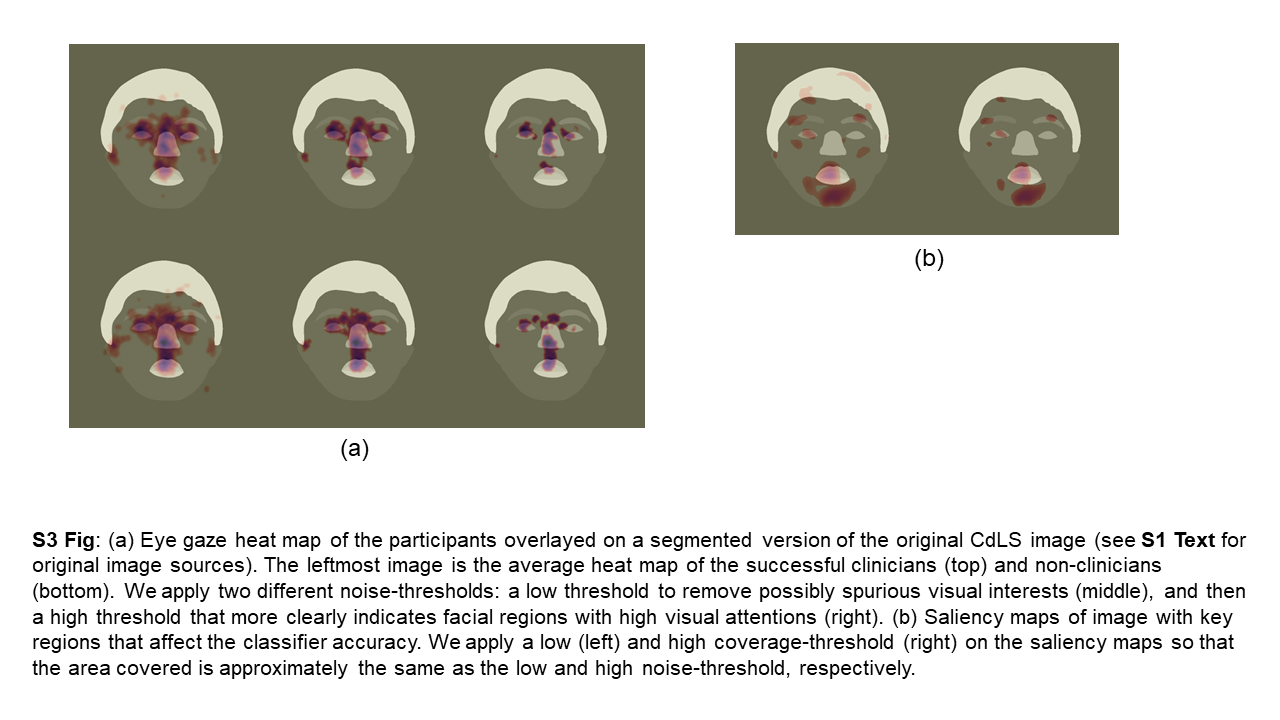

Supplement: S3 Fig — (a) Eye gaze heat map of the participants overlayed on a segmented version of the original CdLS image (see S1 Text for original image sources). The leftmost image is the average heat map of the successful clinicians (top) and non-clinicians (bottom). We apply two different noise-thresholds: a low threshold to remove possibly spurious visual interests (middle), and then a high threshold that more clearly indicates facial regions with high visual attentions (right). (b) Saliency maps of image with key regions that affect the classifier accuracy. We apply a low (left) and high coverage-threshold (right) on the saliency maps so that the area covered is approximately the same as the low and high noise-threshold, respectively. (TIF) [file pgen.1011168.s003.tif]

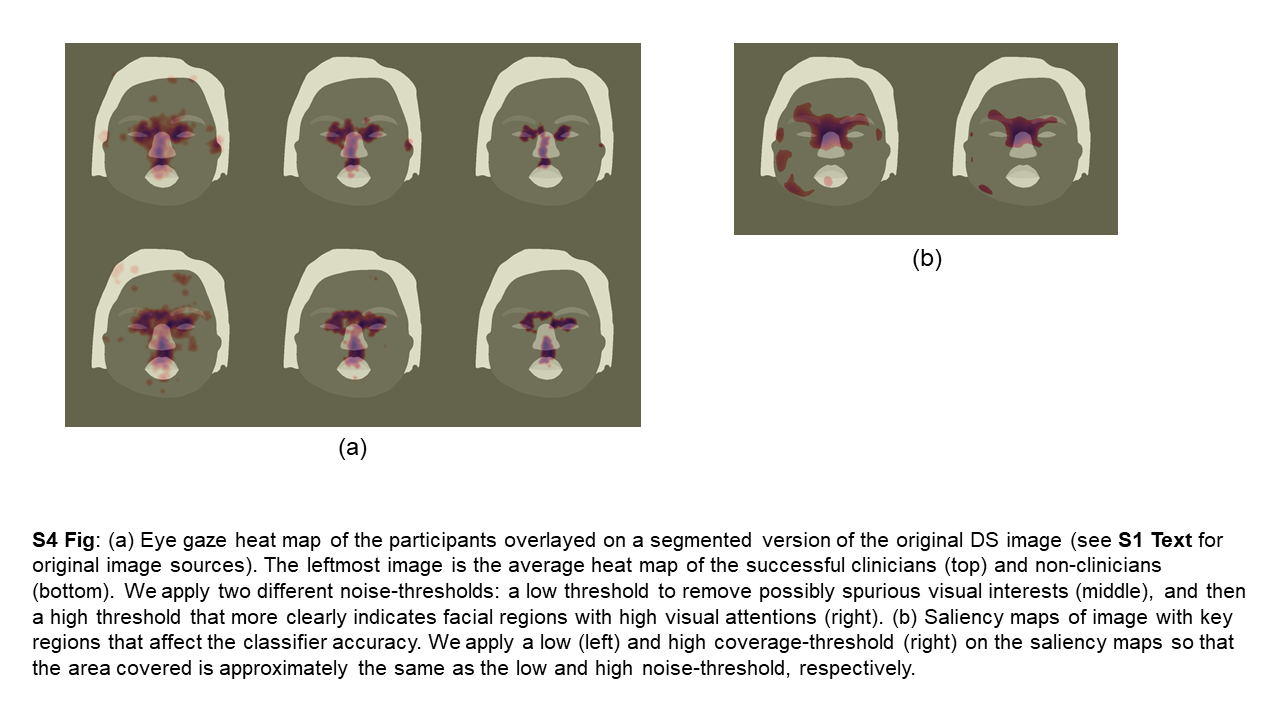

Supplement: S4 Fig — (a) Eye gaze heat map of the participants overlayed on a segmented version of the original DS image (see S1 Text for original image sources). The leftmost image is the average heat map of the successful clinicians (top) and non-clinicians (bottom). We apply two different noise-thresholds: a low threshold to remove possibly spurious visual interests (middle), and then a high threshold that more clearly indicates facial regions with high visual attentions (right). (b) Saliency maps of image with key regions that affect the classifier accuracy. We apply a low (left) and high coverage-threshold (right) on the saliency maps so that the area covered is approximately the same as the low and high noise-threshold, respectively. (TIF) [file pgen.1011168.s004.tif]

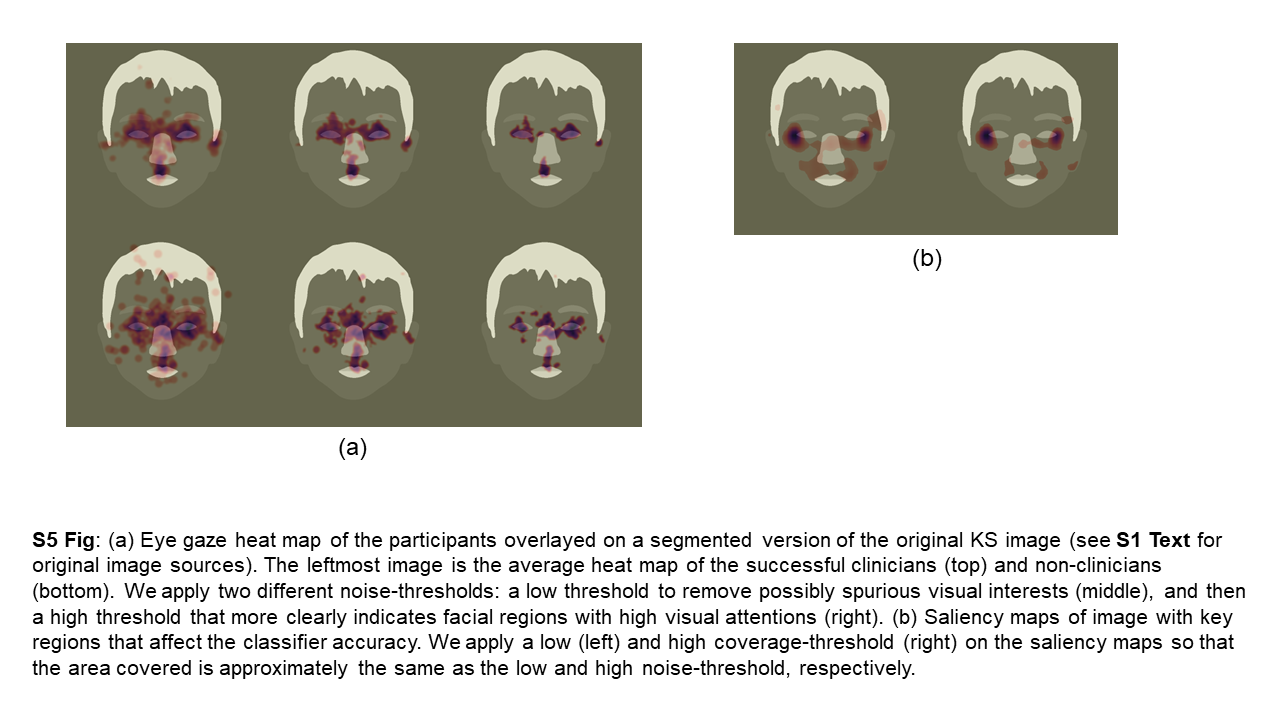

Supplement: S5 Fig — (a) Eye gaze heat map of the participants overlayed on a segmented version of the original KS image (see S1 Text for original image sources). The leftmost image is the average heat map of the successful clinicians (top) and non-clinicians (bottom). We apply two different noise-thresholds: a low threshold to remove possibly spurious visual interests (middle), and then a high threshold that more clearly indicates facial regions with high visual attentions (right). (b) Saliency maps of image with key regions that affect the classifier accuracy. We apply a low (left) and high coverage-threshold (right) on the saliency maps so that the area covered is approximately the same as the low and high noise-threshold, respectively. (TIF) [file pgen.1011168.s005.tif]

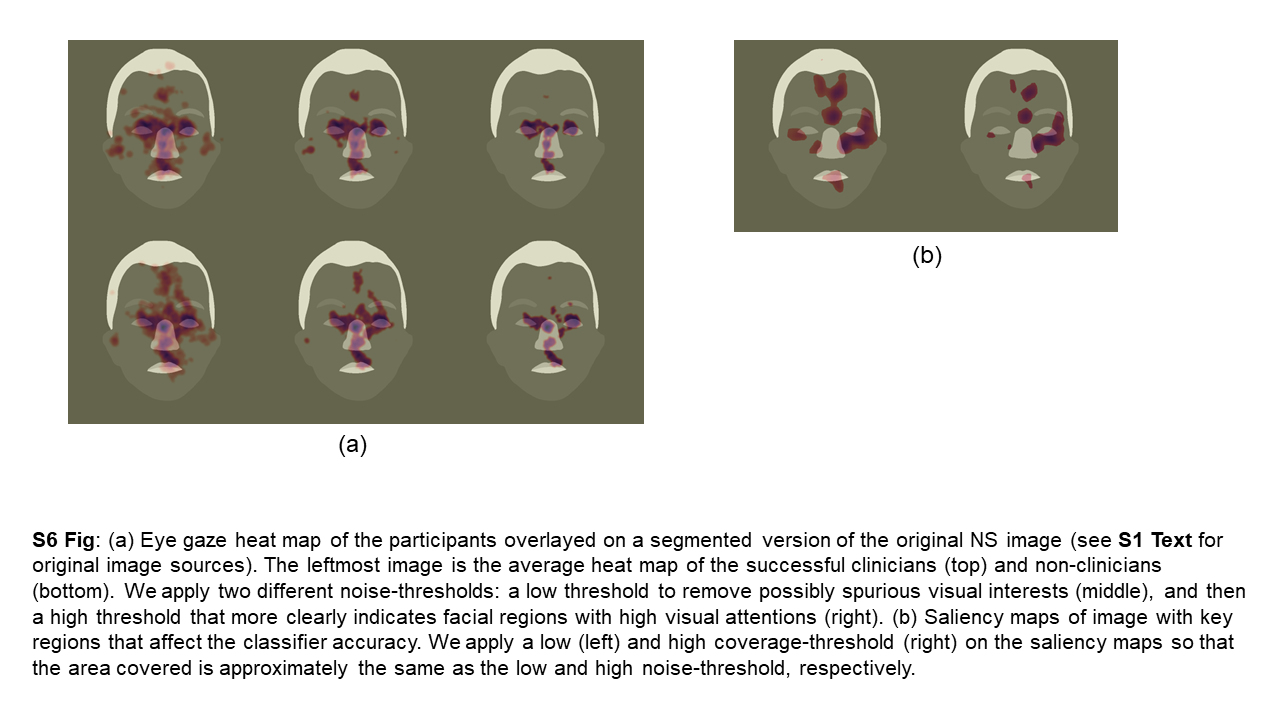

Supplement: S6 Fig — (a) Eye gaze heat map of the participants overlayed on a segmented version of the original NS image (see S1 Text for original image sources). The leftmost image is the average heat map of the successful clinicians (top) and non-clinicians (bottom). We apply two different noise-thresholds: a low threshold to remove possibly spurious visual interests (middle), and then a high threshold that more clearly indicates facial regions with high visual attentions (right). (b) Saliency maps of image with key regions that affect the classifier accuracy. We apply a low (left) and high coverage-threshold (right) on the saliency maps so that the area covered is approximately the same as the low and high noise-threshold, respectively. (TIF) [file pgen.1011168.s006.tif]

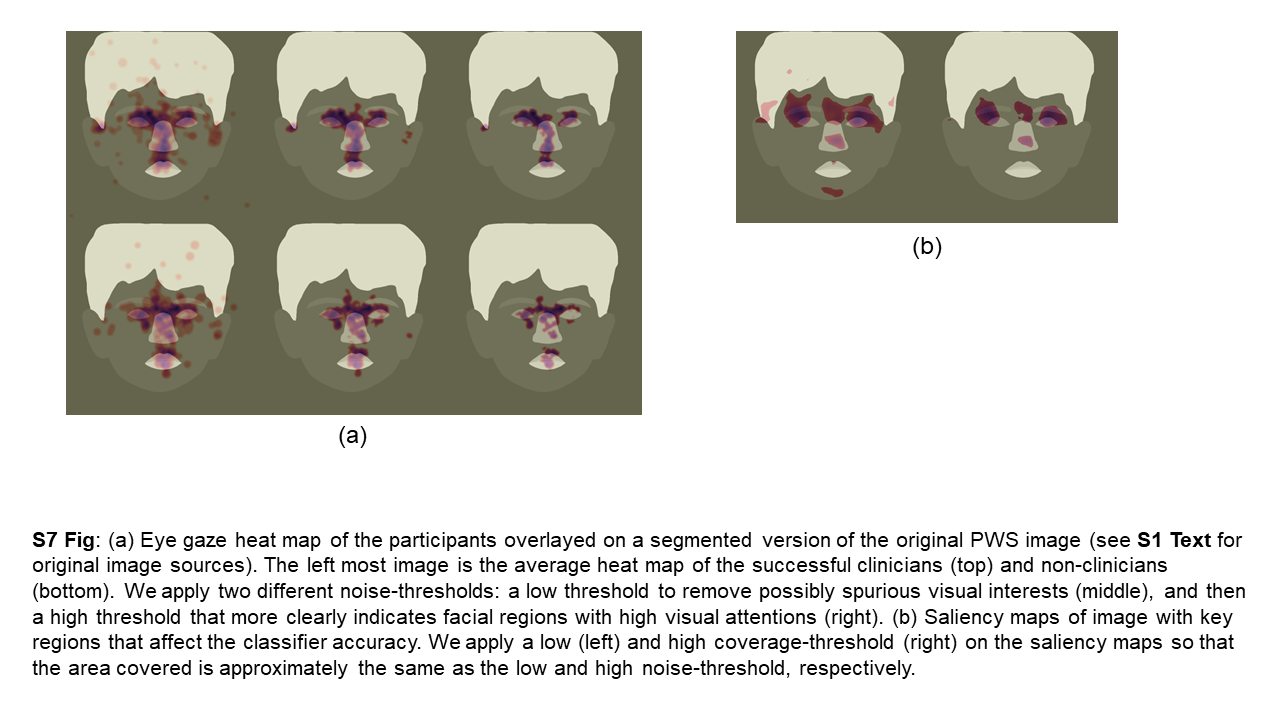

Supplement: S7 Fig — (a) Eye gaze heat map of the participants overlayed on a segmented version of the original PWS image (see S1 Text for original image sources). The leftmost image is the average heat map of the successful clinicians (top) and non-clinicians (bottom). We apply two different noise-thresholds: a low threshold to remove possibly spurious visual interests (middle), and then a high threshold that more clearly indicates facial regions with high visual attentions (right). (b) Saliency maps of image with key regions that affect the classifier accuracy. We apply a low (left) and high coverage-threshold (right) on the saliency maps so that the area covered is approximately the same as the low and high noise-threshold, respectively. (TIF) [file pgen.1011168.s007.tif]

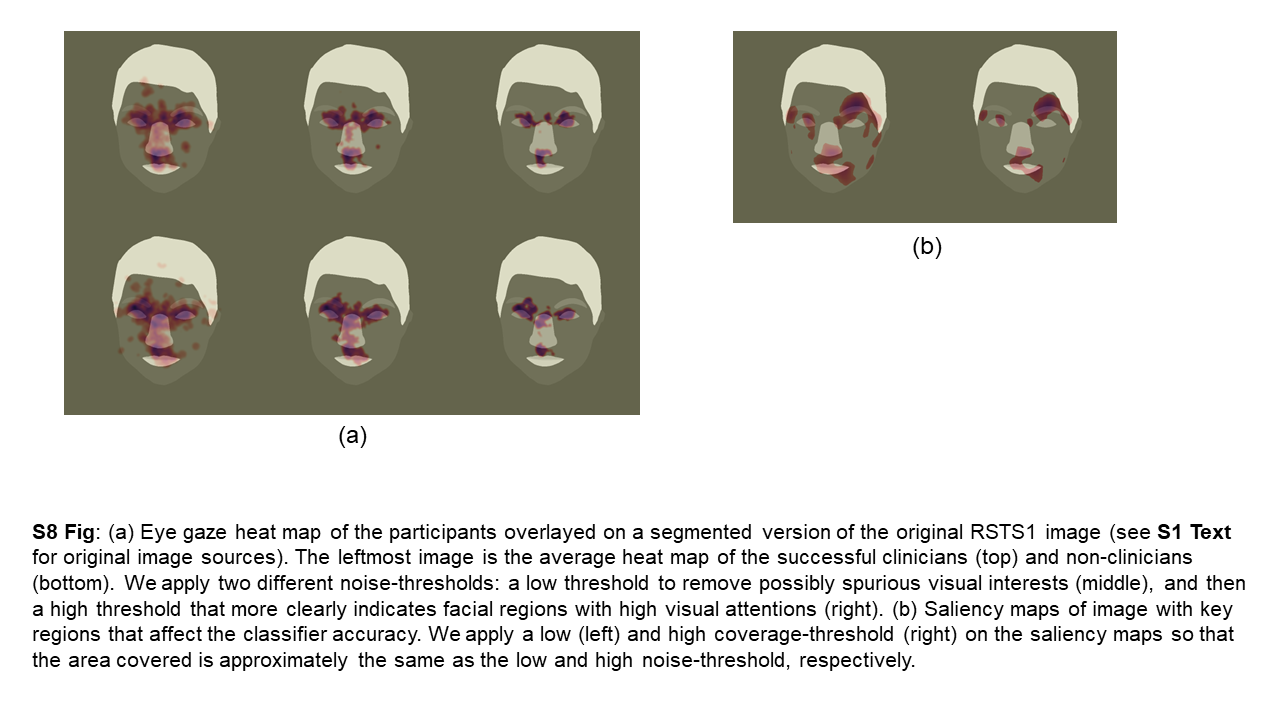

Supplement: S8 Fig — (a) Eye gaze heat map of the participants overlayed on a segmented version of the original RSTS1 image (see S1 Text for original image sources). The leftmost image is the average heat map of the successful clinicians (top) and non-clinicians (bottom). We apply two different noise-thresholds: a low threshold to remove possibly spurious visual interests (middle), and then a high threshold that more clearly indicates facial regions with high visual attentions (right). (b) Saliency maps of image with key regions that affect the classifier accuracy. We apply a low (left) and high coverage-threshold (right) on the saliency maps so that the area covered is approximately the same as the low and high noise-threshold, respectively. (TIF) [file pgen.1011168.s008.tif]

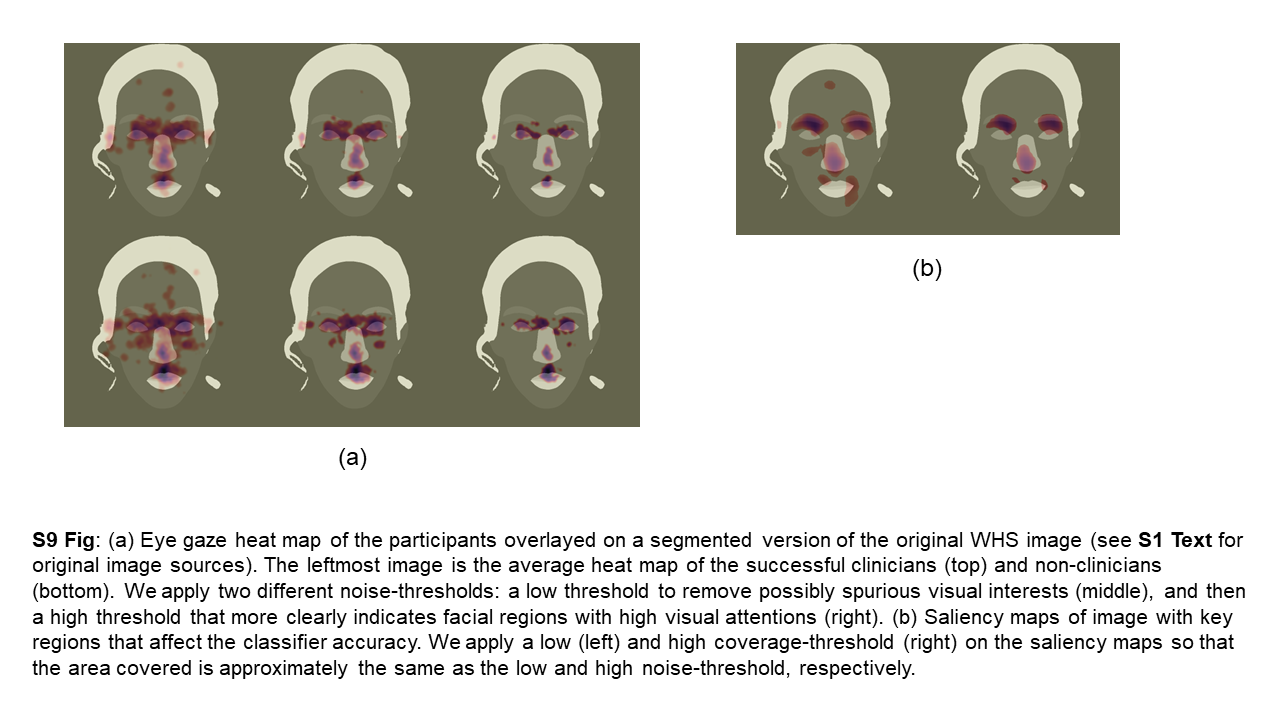

Supplement: S9 Fig — (a) Eye gaze heat map of the participants overlayed on a segmented version of the original WHS image (see S1 Text for original image sources). The leftmost image is the average heat map of the successful clinicians (top) and non-clinicians (bottom). We apply two different noise-thresholds: a low threshold to remove possibly spurious visual interests (middle), and then a high threshold that more clearly indicates facial regions with high visual attentions (right). (b) Saliency maps of image with key regions that affect the classifier accuracy. We apply a low (left) and high coverage-threshold (right) on the saliency maps so that the area covered is approximately the same as the low and high noise-threshold, respectively. (TIF) [file pgen.1011168.s009.tif]

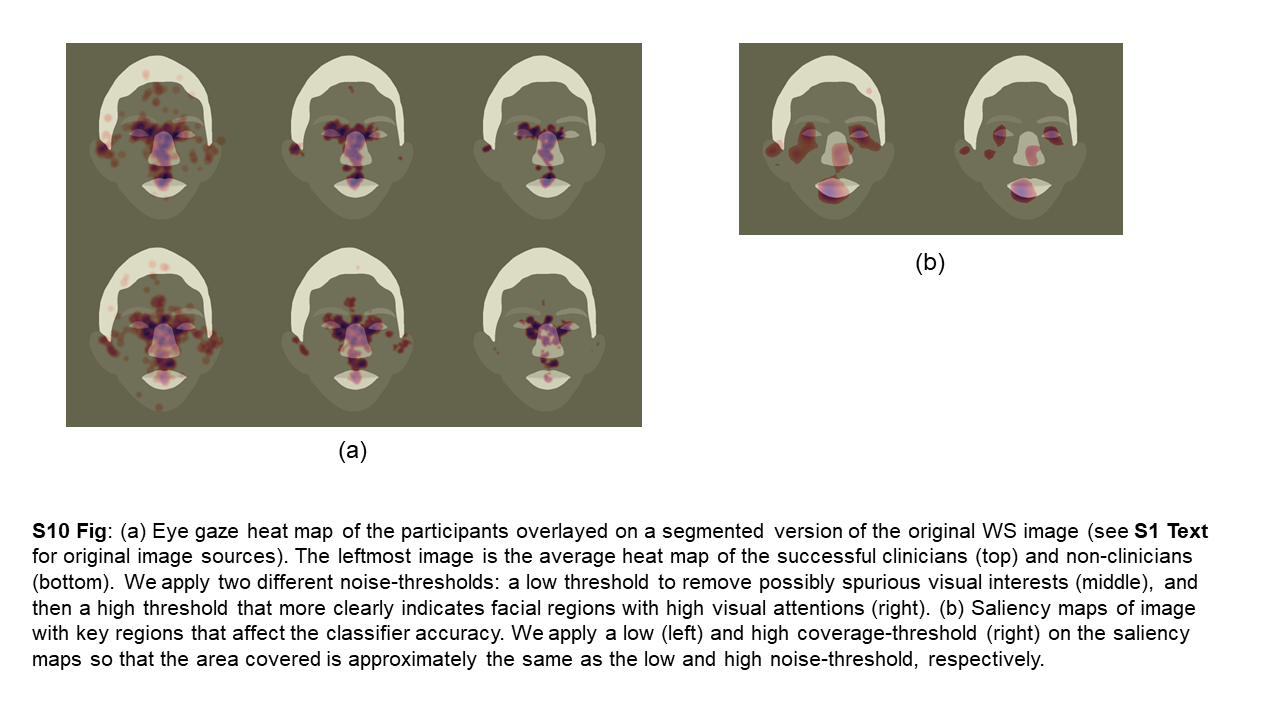

Supplement: S10 Fig — (a) Eye gaze heat map of the participants overlayed on a segmented version of the original WS image (see S1 Text for original image sources). The leftmost image is the average heat map of the successful clinicians (top) and non-clinicians (bottom). We apply two different noise-thresholds: a low threshold to remove possibly spurious visual interests (middle), and then a high threshold that more clearly indicates facial regions with high visual attentions (right). (b) Saliency maps of image with key regions that affect the classifier accuracy. We apply a low (left) and high coverage-threshold (right) on the saliency maps so that the area covered is approximately the same as the low and high noise-threshold, respectively. (TIF) [file pgen.1011168.s010.tif]

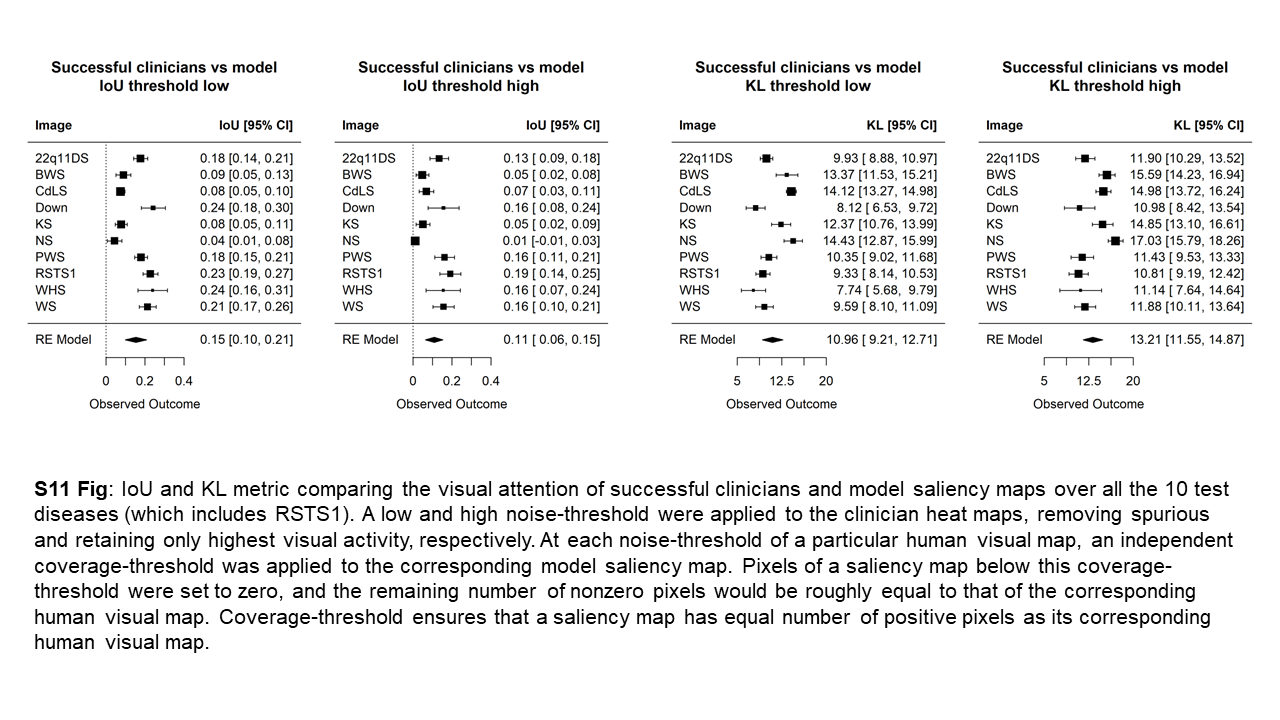

Supplement: S11 Fig — IoU and KL metric comparing the visual attention of successful clinicians and model saliency maps over all the 10 test diseases (which includes RSTS1). A low and high noise-threshold were applied to the clinician heat maps, removing spurious and retaining only highest visual activity, respectively. At each noise-threshold of a particular human visual map, an independent coverage-threshold was applied to the corresponding model saliency map. Pixels of a saliency map below this coverage-threshold were set to zero, and the remaining number of nonzero pixels would be roughly equal to that of the corresponding human visual map. Coverage-threshold ensures that a saliency map has equal number of positive pixels as its corresponding human visual map. (TIF) [file pgen.1011168.s011.tif]

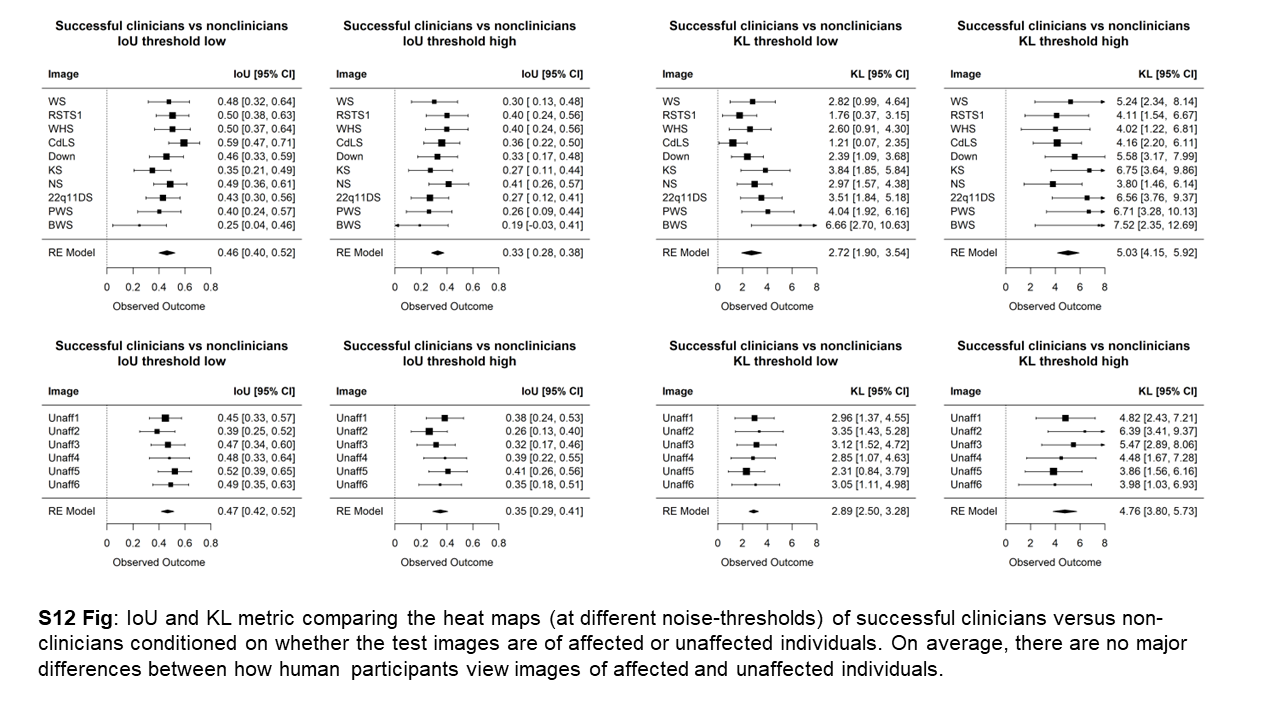

Supplement: S12 Fig — IoU and KL metric comparing the heat maps (at different noise-threshold) of successful clinicians versus non-clinicians conditioned on whether the test images are of affected or unaffected individuals. On average, there is no major differences between how human participants view images of affected and unaffected individuals. (TIF) [file pgen.1011168.s012.tif]

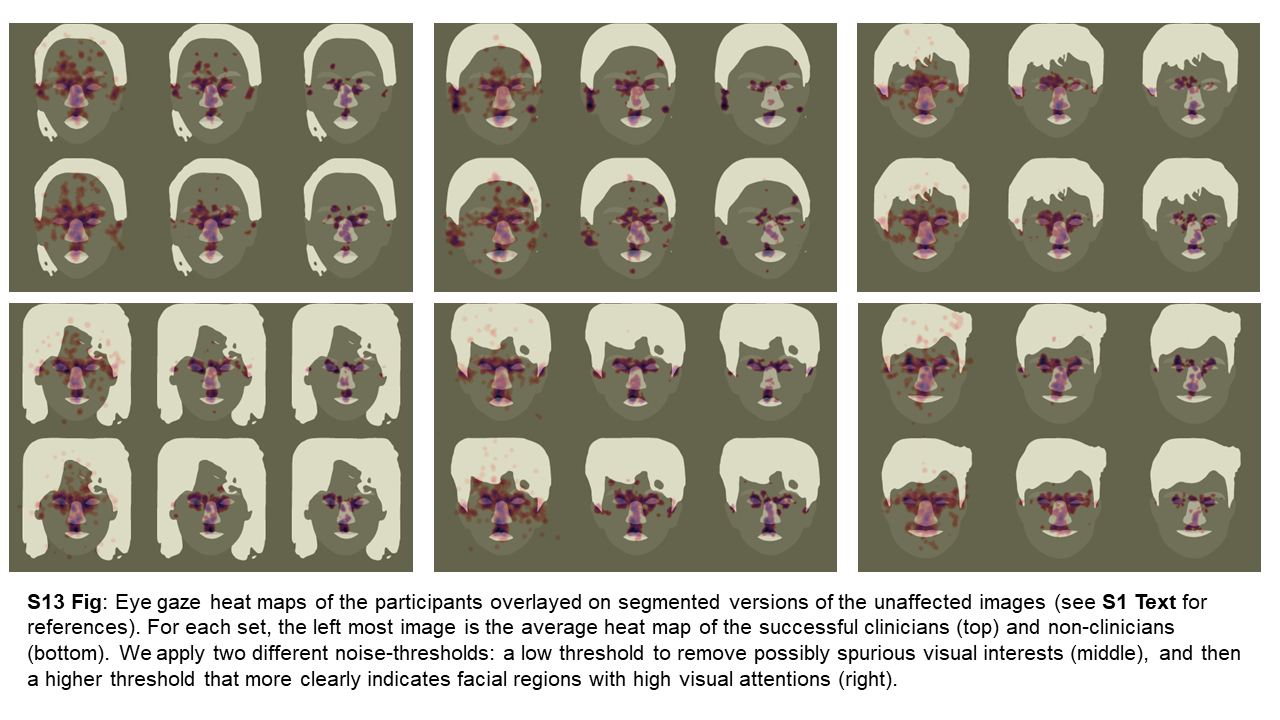

Supplement: S13 Fig — Eye gaze heat map of the participants overlayed on the unaffected images. For each set, the left most image is the average heat map of the successful clinicians (top) and non-clinicians (bottom). We apply two different noise-thresholds: a low threshold to remove possibly spurious visual interests (middle), and then a higher threshold that more clearly indicates facial regions with high visual attentions (right). (TIF) [file pgen.1011168.s013.tif]

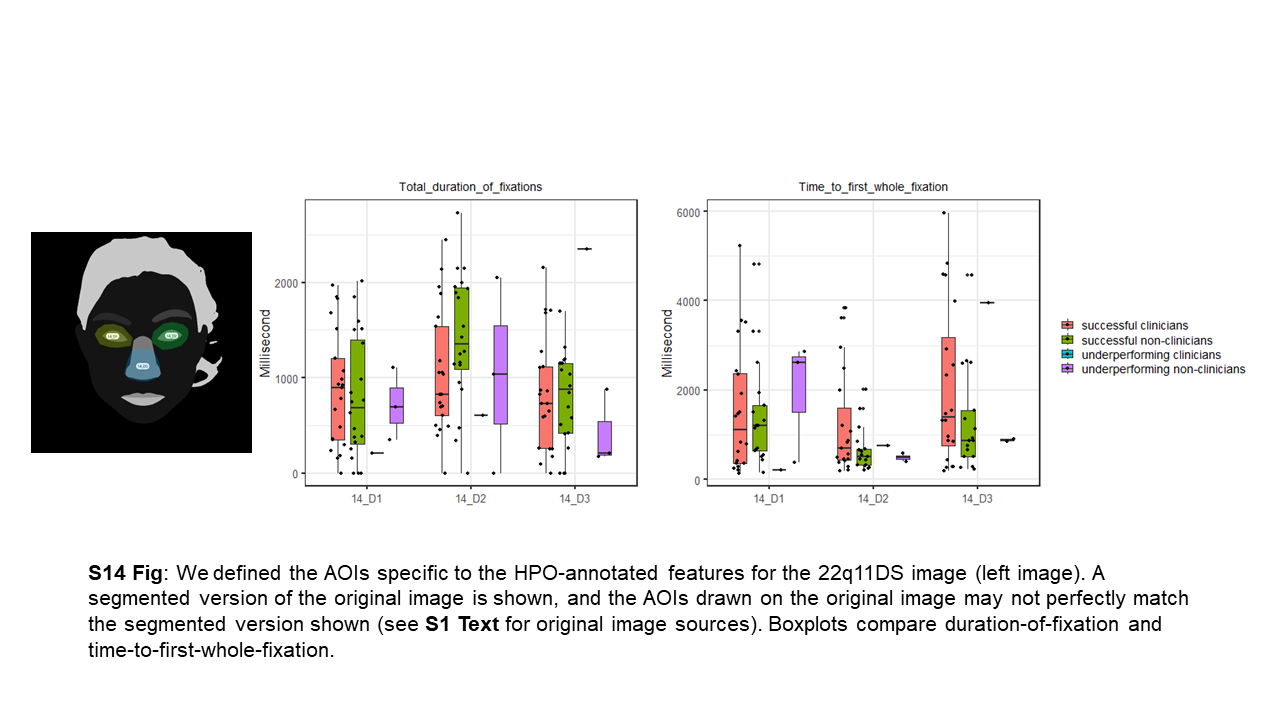

Supplement: S14 Fig — We defined the AOIs specific to the HPO-annotated features for the 22q11DS image (left image). A segmented version of the original image is shown, and the AOIs drawn on the original image may not perfectly match the segmented version shown (see S1 Text for original image sources). Boxplots compare duration-of-fixation and time-to-first-whole-fixation. (TIF) [file pgen.1011168.s014.tif]

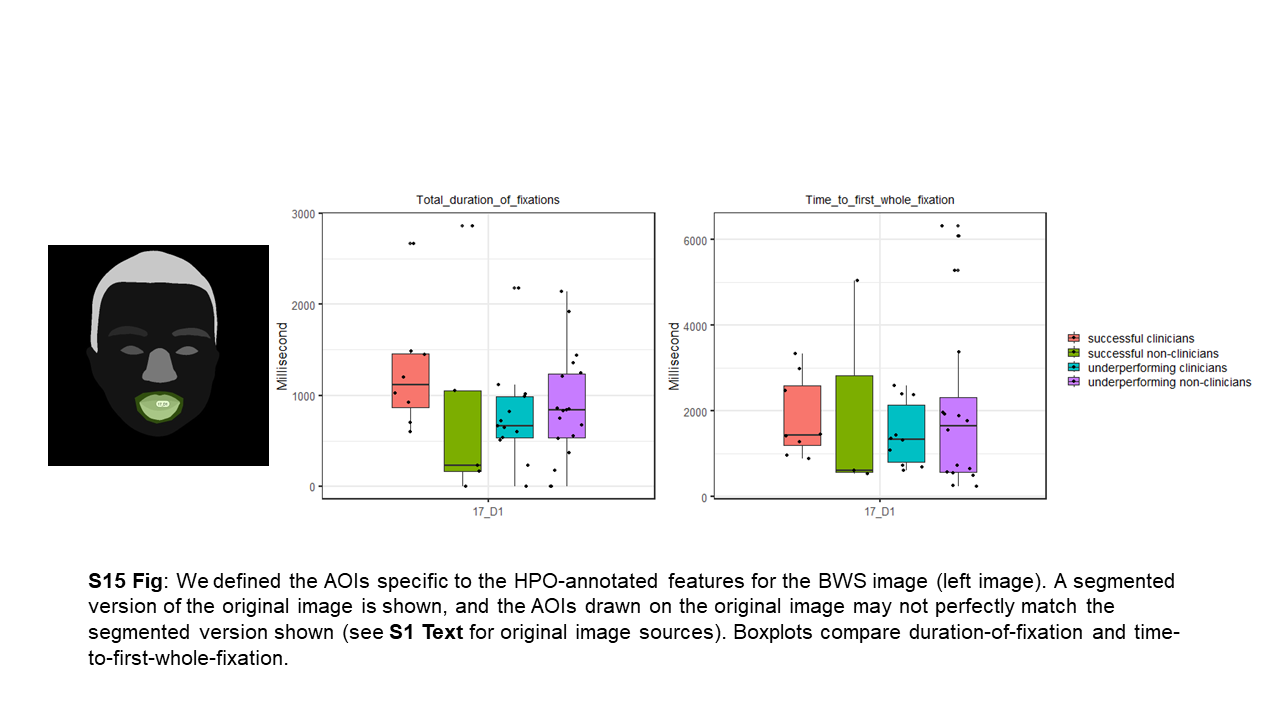

Supplement: S15 Fig — We defined the AOIs specific to the HPO-annotated features for the BWS image (left image). A segmented version of the original image is shown, and the AOIs drawn on the original image may not perfectly match the segmented version shown (see S1 Text for original image sources). Boxplots compare duration-of-fixation and time-to-first-whole-fixation. (TIF) [file pgen.1011168.s015.tif]

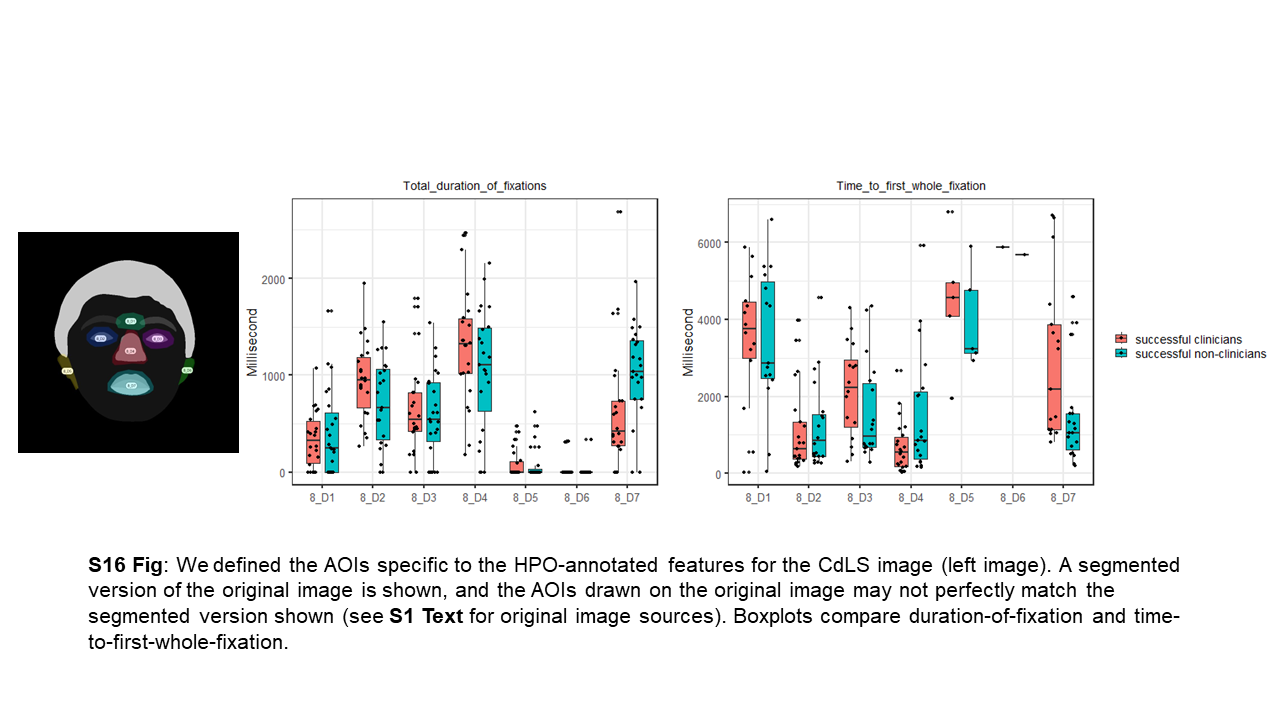

Supplement: S16 Fig — We defined the AOIs specific to the HPO-annotated features for the CdLS image (left image). A segmented version of the original image is shown, and the AOIs drawn on the original image may not perfectly match the segmented version shown (see S1 Text for original image sources). Boxplots compare duration-of-fixation and time-to-first-whole-fixation. (TIF) [file pgen.1011168.s016.tif]

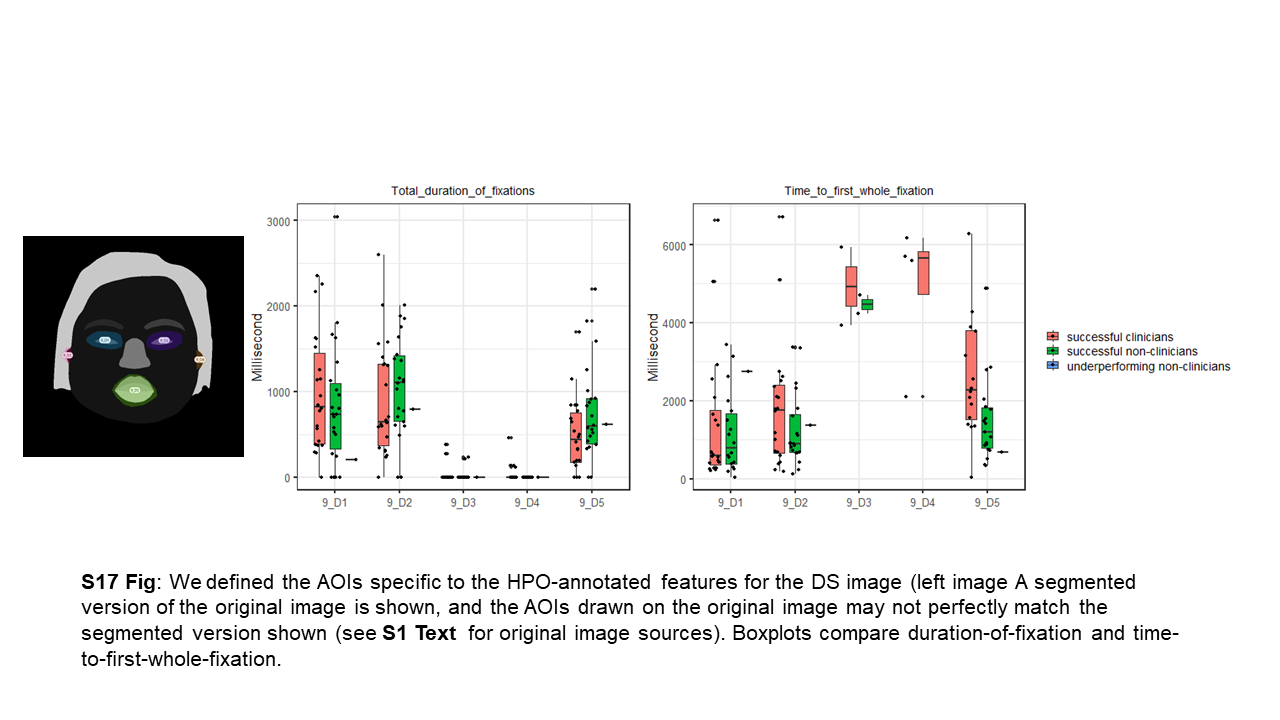

Supplement: S17 Fig — We defined the AOIs specific to the HPO-annotated features for the DS image (left image). A segmented version of the original image is shown, and the AOIs drawn on the original image may not perfectly match the segmented version shown (see S1 Text for original image sources). Boxplots compare duration-of-fixation and time-to-first-whole-fixation. (TIF) [file pgen.1011168.s017.tif]

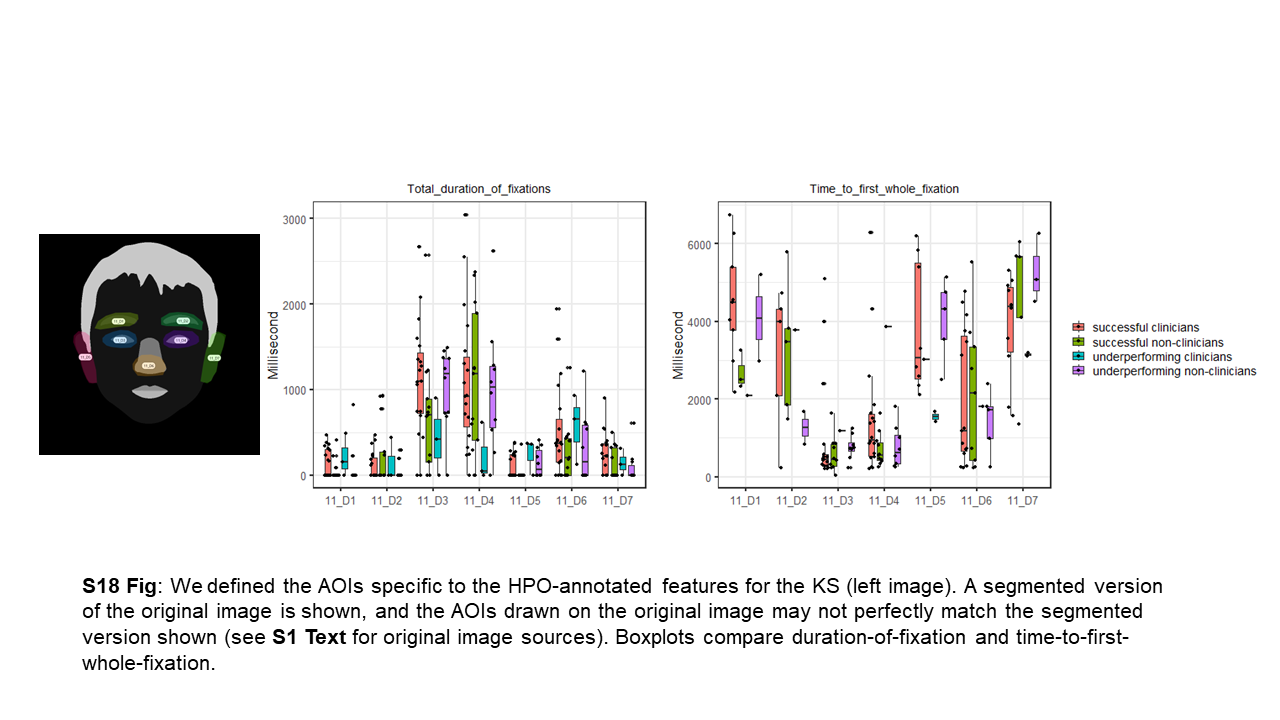

Supplement: S18 Fig — We defined the AOIs specific to the HPO-annotated features for the KS image (left image). A segmented version of the original image is shown, and the AOIs drawn on the original image may not perfectly match the segmented version shown (see S1 Text for original image sources). Boxplots compare duration-of-fixation and time-to-first-whole-fixation. (TIF) [file pgen.1011168.s018.tif]

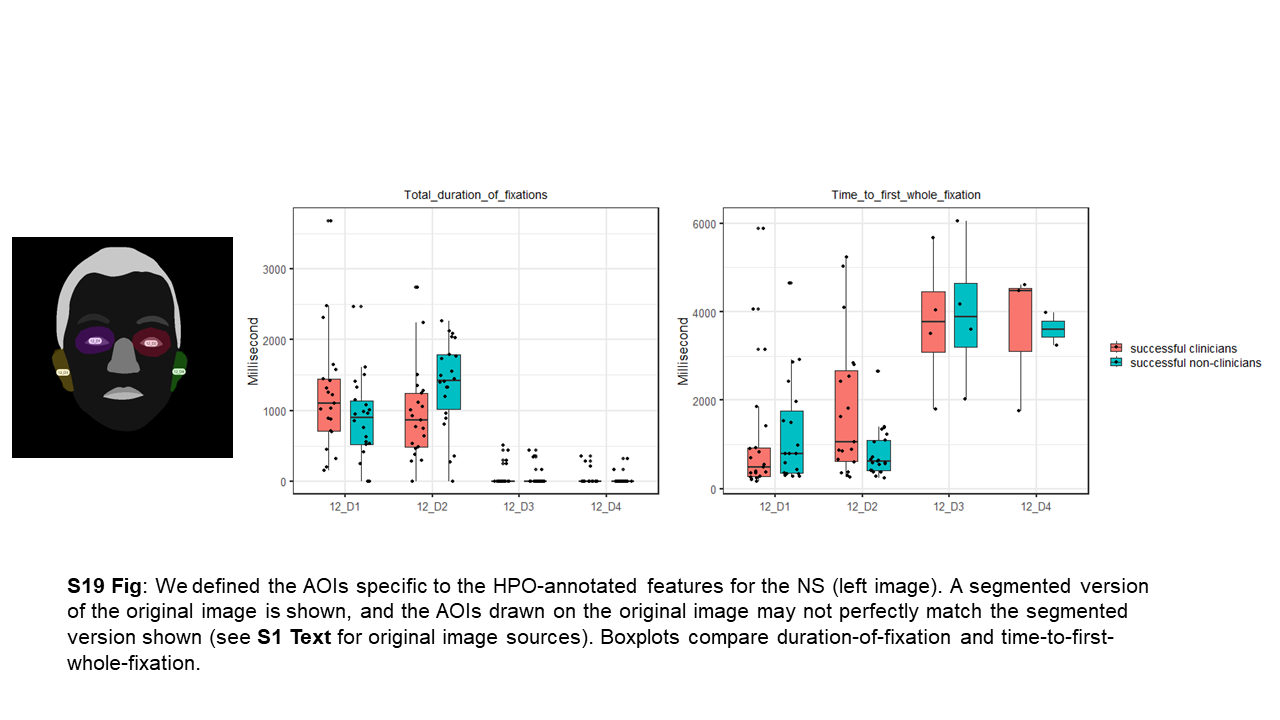

Supplement: S19 Fig — We defined the AOIs specific to the HPO-annotated features for the NS image (left image). A segmented version of the original image is shown, and the AOIs drawn on the original image may not perfectly match the segmented version shown (see S1 Text for original image sources). Boxplots compare duration-of-fixation and time-to-first-whole-fixation. (TIF) [file pgen.1011168.s019.tif]

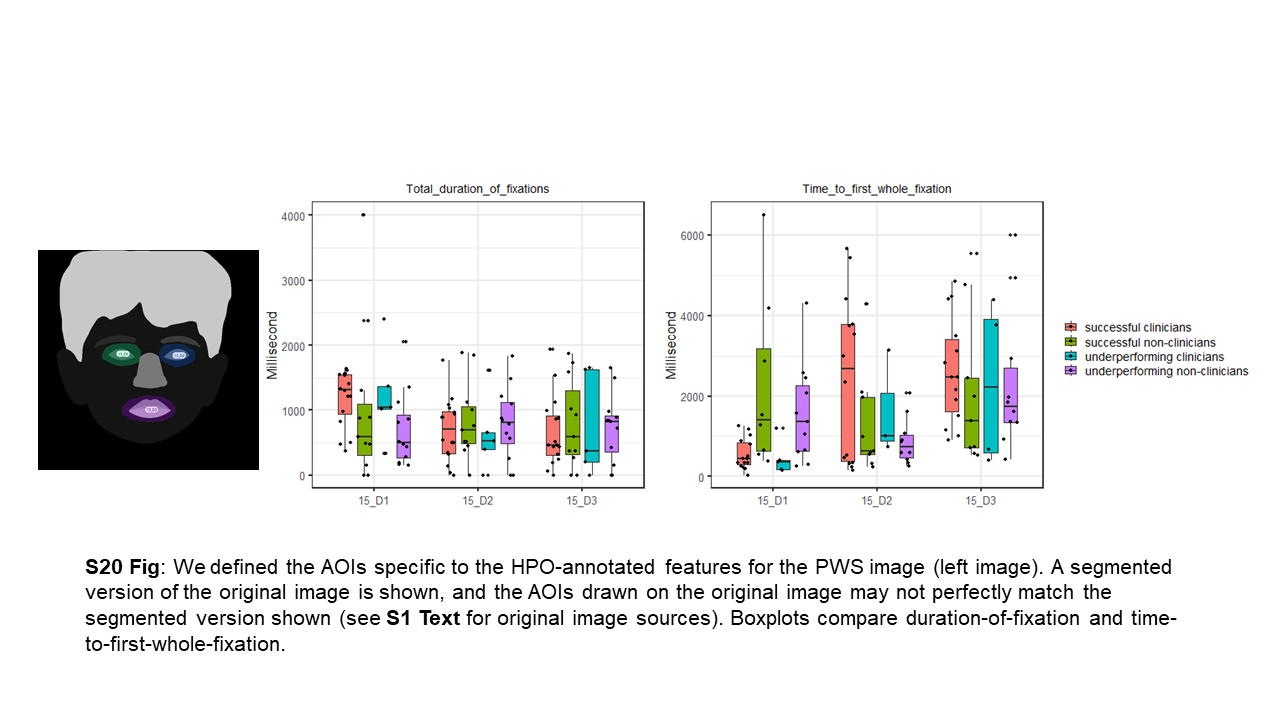

Supplement: S20 Fig — We defined the AOIs specific to the HPO-annotated features for the PWS image (left image). A segmented version of the original image is shown, and the AOIs drawn on the original image may not perfectly match the segmented version shown (see S1 Text for original image sources). Boxplots compare duration-of-fixation and time-to-first-whole-fixation. (TIF) [file pgen.1011168.s020.tif]

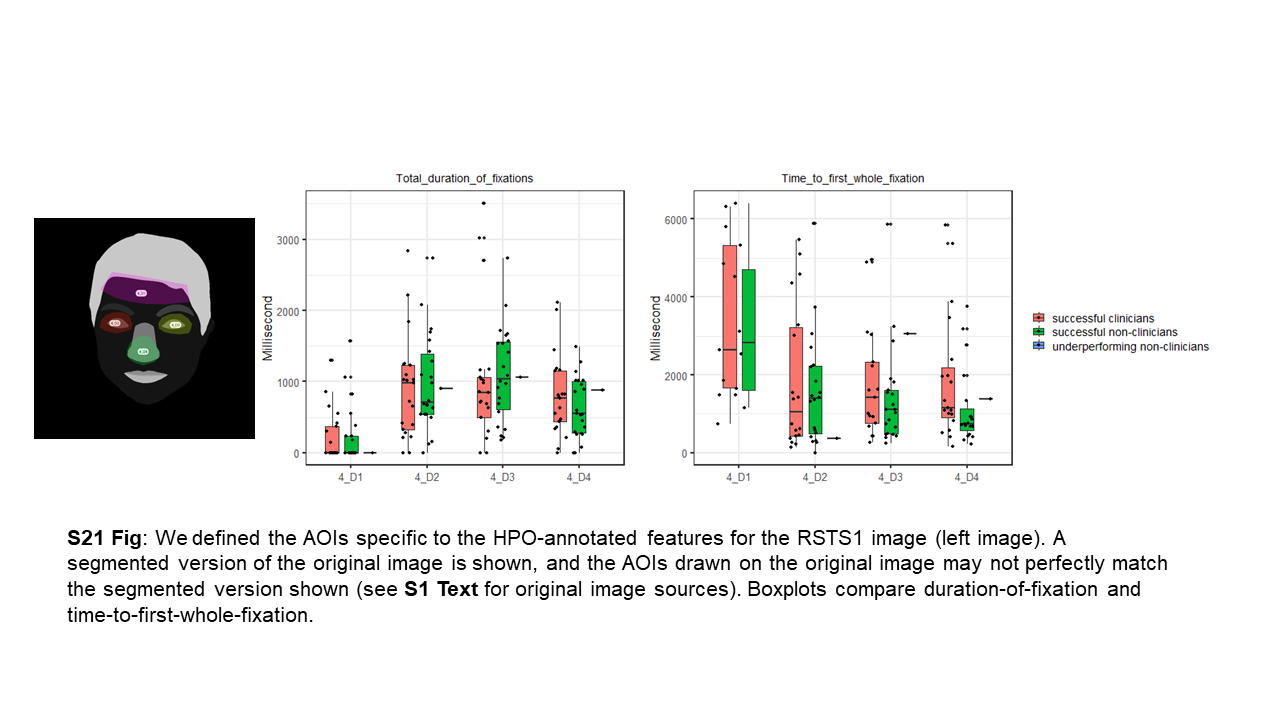

Supplement: S21 Fig — We defined the AOIs specific to the HPO-annotated features for the RSTS1 image (left image). A segmented version of the original image is shown, and the AOIs drawn on the original image may not perfectly match the segmented version shown (see S1 Text for original image sources). Boxplots compare duration-of-fixation and time-to-first-whole-fixation. (TIF) [file pgen.1011168.s021.tif]

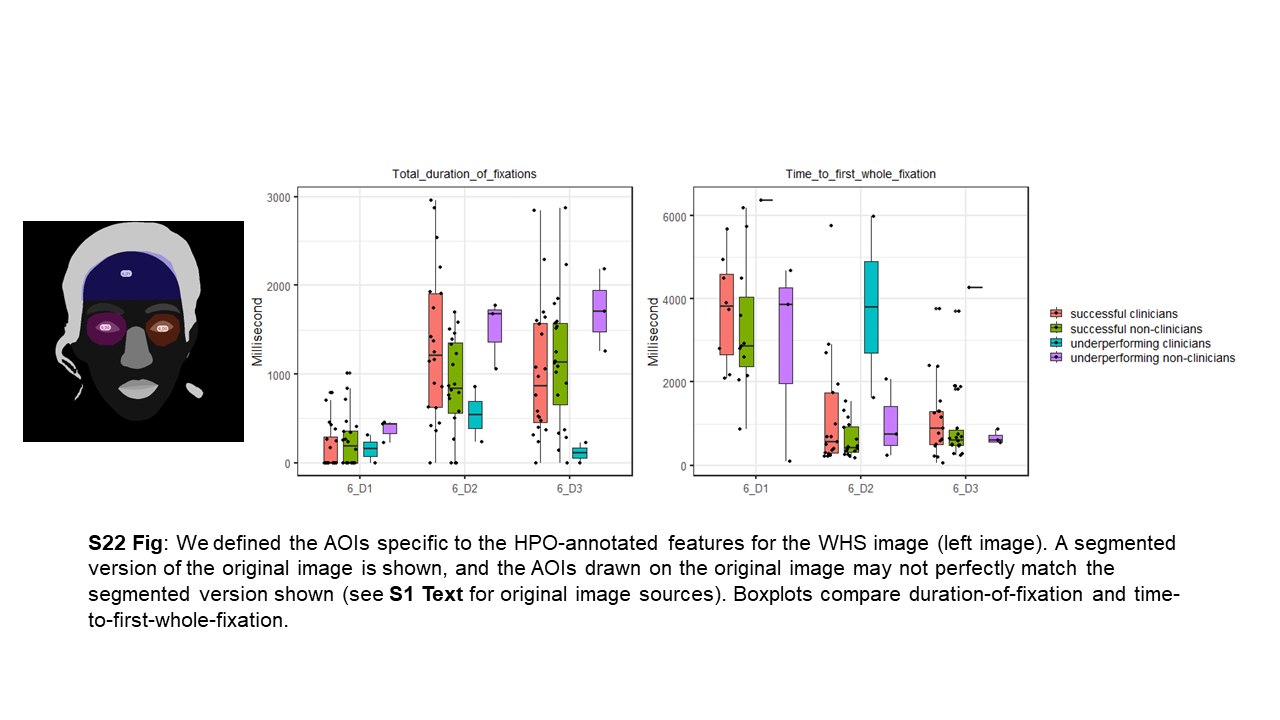

Supplement: S22 Fig — We defined the AOIs specific to the HPO-annotated features for the WHS image (left image). A segmented version of the original image is shown, and the AOIs drawn on the original image may not perfectly match the segmented version shown (see S1 Text for original image sources). Boxplots compare duration-of-fixation and time-to-first-whole-fixation. (TIF) [file pgen.1011168.s022.tif]

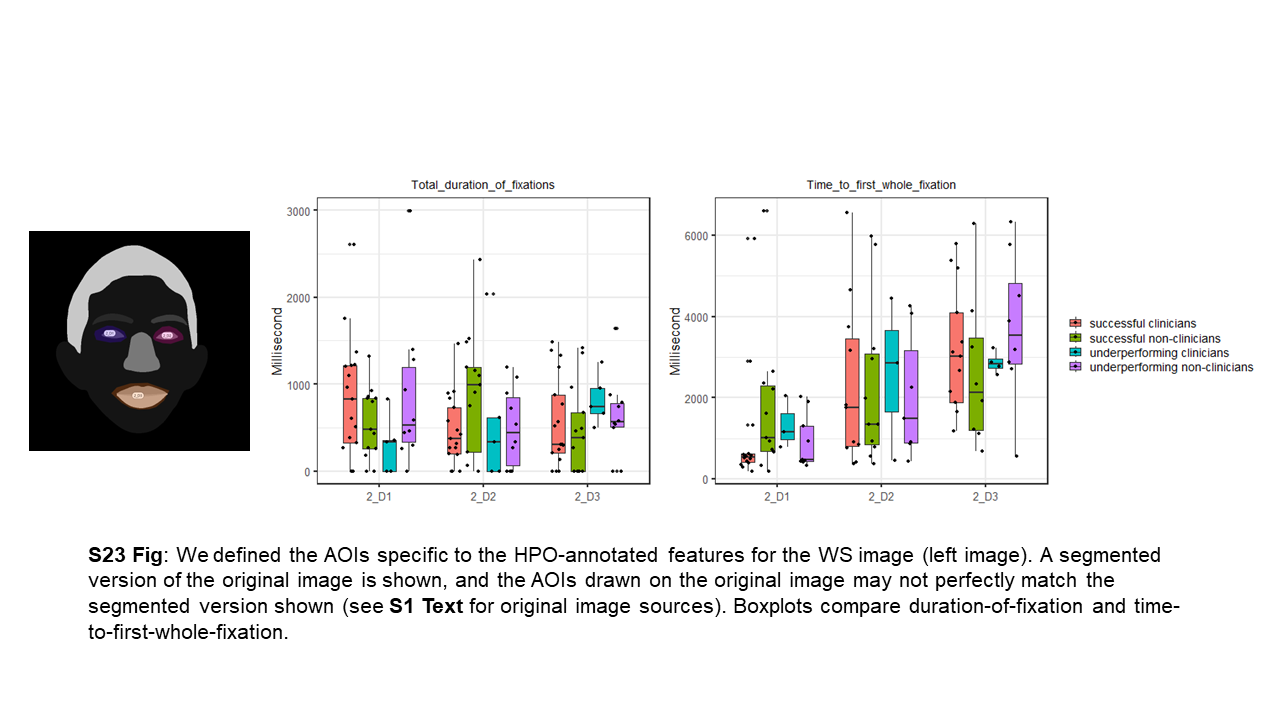

Supplement: S23 Fig — We defined the AOIs specific to the HPO-annotated features for the WS image (left image). A segmented version of the original image is shown, and the AOIs drawn on the original image may not perfectly match the segmented version shown (see S1 Text for original image sources). Boxplots compare duration-of-fixation and time-to-first-whole-fixation. (TIF) [file pgen.1011168.s023.tif]

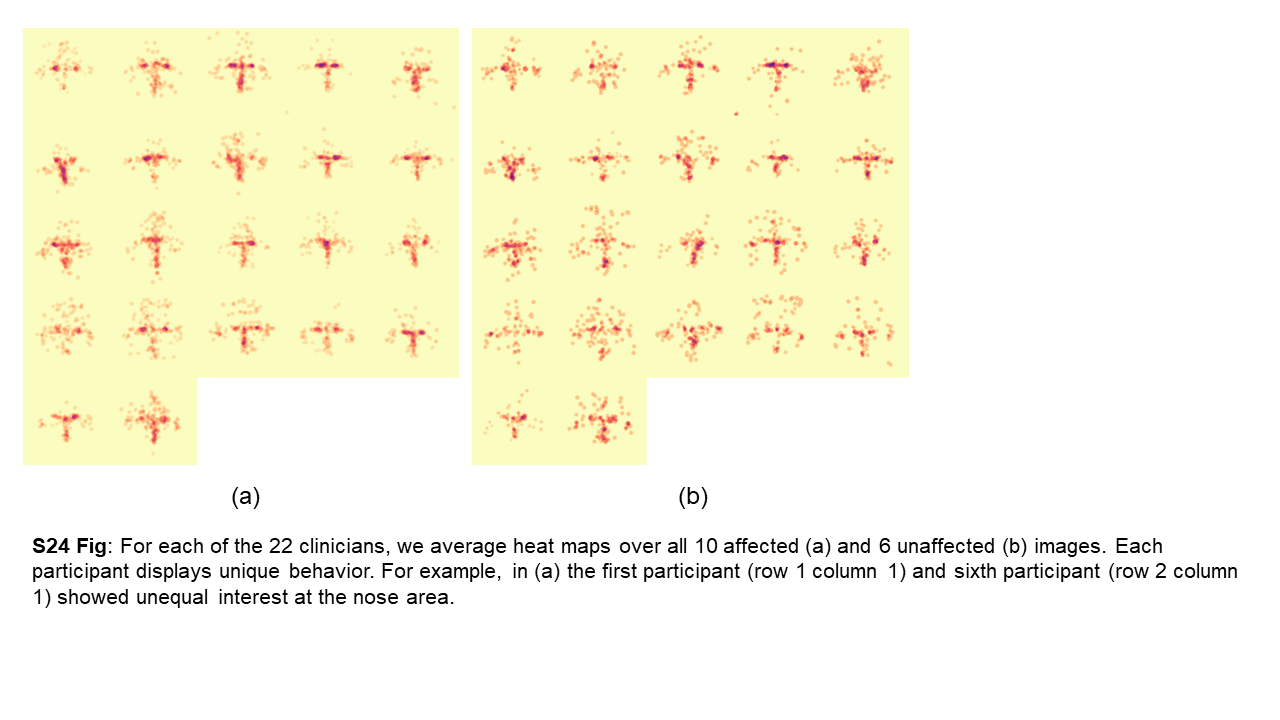

Supplement: S24 Fig — For each of the 22 clinicians, we average heat maps over all 10 affected (a) and 6 unaffected (b) images. Each participant displays unique behavior. For example, in (a) the first participant (row 1 column 1) and sixth participant (row 2 column 1) showed unequal interest at the nose area. (TIF) [file pgen.1011168.s024.tif]

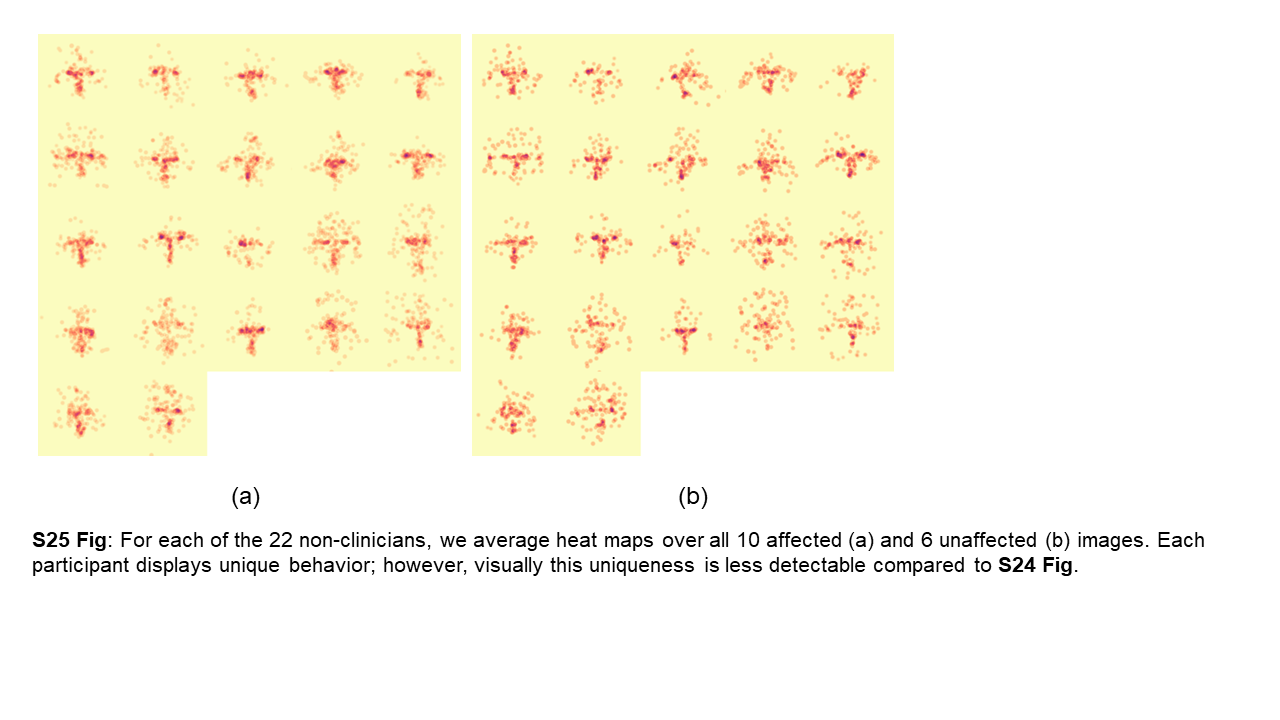

Supplement: S25 Fig — For each of the 22 non-clinicians, we average heat maps over all 10 affected (a) and 6 unaffected (b) images. Each participant displays unique behavior; however, visually this uniqueness is less detectable compared to S24 Fig. (TIF) [file pgen.1011168.s025.tif]

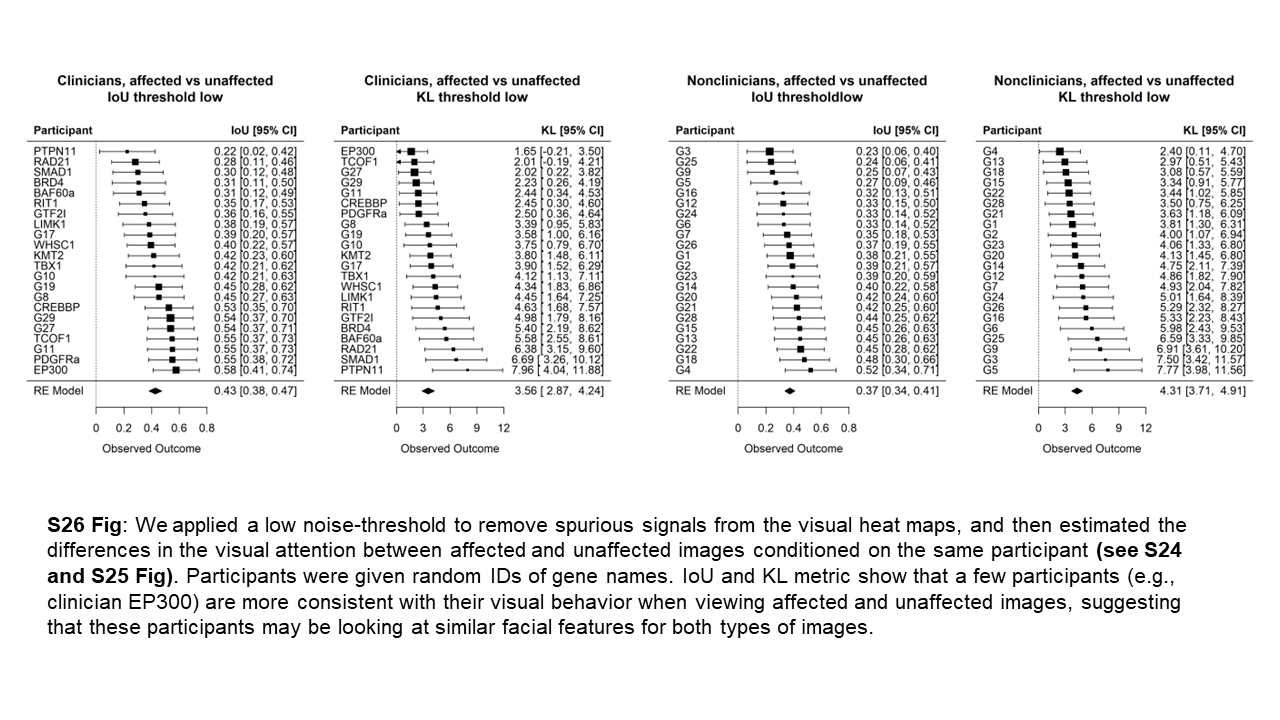

Supplement: S26 Fig — We applied a low noise-threshold to remove spurious signals from the visual heat maps, and then estimated the differences in the visual attention between affected and unaffected images conditioned on the same participant (see S24 and S25 Figs). Participants were given random IDs of gene names. IoU and KL metric show that a few participants (e.g., clinician EP300) are more consistent with their visual behavior when viewing affected and unaffected images, suggesting that these participants may be looking at similar facial features for both types of images. (TIF) [file pgen.1011168.s026.tif]

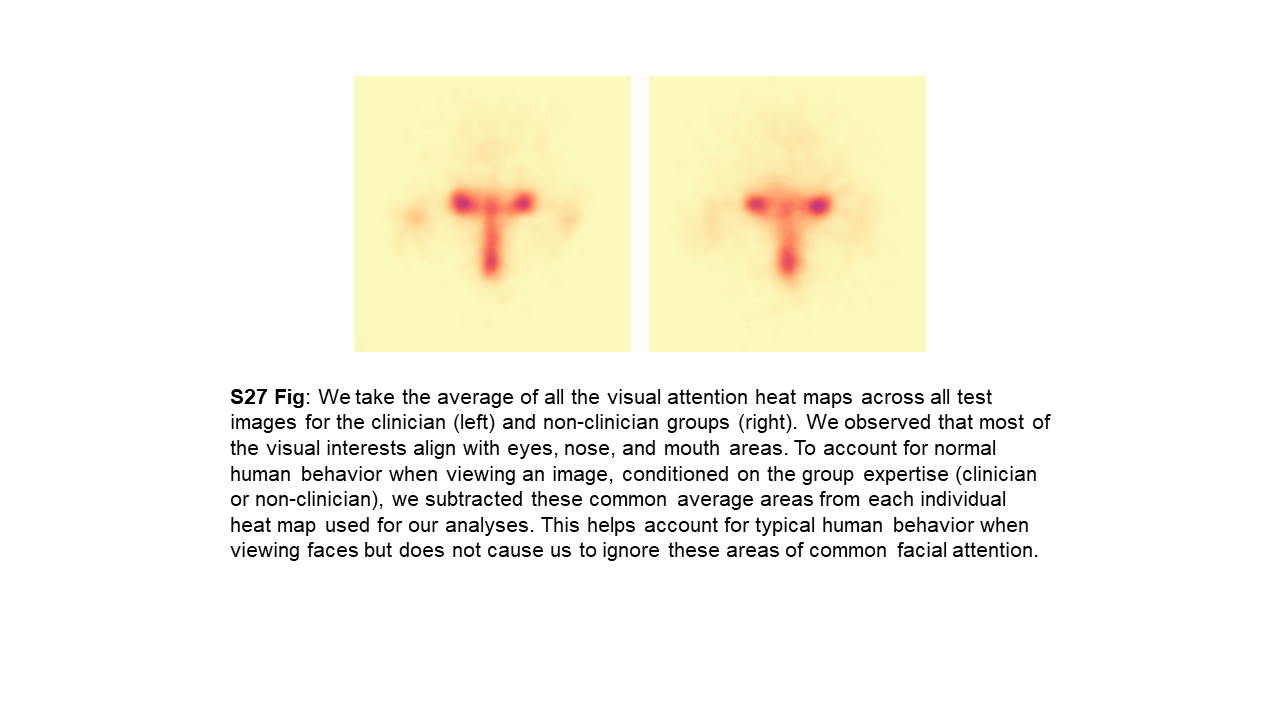

Supplement: S27 Fig — We take the average of all the visual attention heat maps across all test images for the clinician (left) and non-clinician groups (right). We observed that most of the visual interests align with eyes, nose, and mouth areas. To account for normal human behavior when viewing an image, conditioned on the group expertise (clinician or non-clinician), we subtracted these common average areas from each individual heat map used for our analyses. This helps account for typical human behavior when viewing faces but does not cause us to ignore these areas of common facial attention. (TIF) [file pgen.1011168.s027.tif]
