# Supplementary material for: Comparison of clinical geneticist and computer visual attention in assessing genetic conditions
Source: PLoS Genet. 2024 Feb 27;20(2):e1011168. doi: 10.1371/journal.pgen.1011168 (PMC10923488; doi:10.1371/journal.pgen.1011168)
Supplement: S1 Text — Contains Tables A-C and additional explanations about the DL model analyses and image sources. (DOCX) [file pgen.1011168.s028.docx]

**S1 Text**

**Table A** shows the top-k accuracy (acc@k) on the validation set when evaluated against the model trained with our best set of hyperparameters. Our purposes were to compare the results of a classifier saliency map to eye-tracking experiments of human participants, but we did not assume that either was superior or correct in terms of which parts of the images were assessed – we expect different classifiers and different participants to yield different results. We also intentionally chose images (in both the training and test sets) with a range of classifier performance (and with a range of difficulty for human recognition based on our review by clinical geneticists), as we did not want to consider only images that were biased towards better performance for either the DL model or human participants. For this reason, we do not expect our model to have very high acc@1 on the validation set; however, the model has much better acc@2 and acc@3 indicating that it performs reasonably well for our experiments. PWS and RSTS1 have the lowest accuracy, indicating that our model has difficulty with these two conditions. These types of results are similar to our and other previous results when studying rare genetic conditions, which intrinsically involves relatively small datasets for each condition category (1-4).

**Table A.** Top-k accuracy (acc@k) on the validation set, we show the mean (and standard deviation) averaging over the 5 folds.

|  | acc@1 | acc@2 | acc@3 | Number of images |
| --- | --- | --- | --- | --- |
| 22q11DS | 0.750 (0.050) | 0.884 (0.027) | 0.938 (0.023) | 591 |
| BWS | 0.620 (0.032) | 0.775 (0.027) | 0.866 (0.030) | 308 |
| CdLS | 0.768 (0.036) | 0.833 (0.008) | 0.908 (0.033) | 120 |
| Down | 0.803 (0.044) | 0.909 (0.022) | 0.942 (0.018) | 352 |
| KS | 0.735 (0.057) | 0.834 (0.042) | 0.901 (0.032) | 246 |
| NS | 0.666 (0.024) | 0.831 (0.014) | 0.899 (0.026) | 325 |
| PWS | 0.388 (0.092) | 0.564 (0.109) | 0.662 (0.103) | 104 |
| RSTS1 | 0.619 (0.108) | 0.741 (0.155) | 0.791 (0.128) | 105 |
| Unaffected | 0.744 (0.092) | 0.875 (0.064) | 0.897 (0.056) | 228 |
| WHS | 0.654 (0.128) | 0.741 (0.109) | 0.830 (0.110) | 178 |
| WS | 0.830 (0.015) | 0.917 (0.029) | 0.949 (0.031) | 531 |

**Table B.** Ground-truth prediction probability for each image used in eye-tracking experiments. Second and third column: For CdLS, PWS, and RSTS1, the model’s highest predicted probabilities correspond to different labels than the ground-truths (hence, the model misclassified). The last two columns indicate whether the correct labels were found within the top-1 (acc@1) and top-2 (acc@2) accuracy.

|  | Ground-truth prediction | Model highest prediction | Label of highest prediction | acc@1 | acc@2 |
| --- | --- | --- | --- | --- | --- |
| 22q11DS | 0.5230 |  |  | 1 |  |
| BWS | 0.4216 |  |  | 1 |  |
| CdLS | 0.3196 | 0.3285 | NS |  | 1 |
| Down | 0.9966 |  |  | 1 |  |
| KS | 0.8822 |  |  | 1 |  |
| NS | 0.6512 |  |  | 1 |  |
| PWS | 0.3383 | 0.4690 | WS |  | 1 |
| RSTS1 | 0.0005 | 0.7976 | NS |  |  |
| Unaffected image 1 | 0.9008 |  |  | 1 |  |
| Unaffected image 2 | 0.4456 |  |  | 1 |  |
| Unaffected image 3 | 0.9599 |  |  | 1 |  |
| Unaffected image 4 | 0.8092 |  |  | 1 |  |
| Unaffected image 5 | 0.8380 |  |  | 1 |  |
| Unaffected image 6 | 0.9859 |  |  | 1 |  |
| WHS | 0.7983 |  |  | 1 |  |
| WS | 0.9960 |  |  | 1 |  |

**Table C.** Ground-truth prediction probability for additional images selected as test set along with images used in eye-tracking experiments. The second and third columns show if the model’s highest predicted probabilities correspond to different labels than the ground-truths (hence, the model misclassified). The last two columns indicate whether the correct labels were found within the top-1 (acc@1), top-2 (acc@2), and top-3 (acc@3) accuracy. See the GitHub links listed under Data and code availability section in the main manuscript.

|  | Ground-truth prediction | Model highest prediction | Label of highest prediction | acc@1 | acc@2 | acc@3 |
| --- | --- | --- | --- | --- | --- | --- |
| 22q11DS image 277.png | 0.9999 |  |  | 1 |  |  |
| 22q11DS image 314.png | 0.6389 |  |  | 1 |  |  |
| BWS image 64.png | 0.3904 | 0.4230 | WS |  | 1 |  |
| BWS image 10.png | 0.1894 | 0.5070 | 22q11DS |  |  | 1 |
| BWS image 11.png | 0.9944 |  |  | 1 |  |  |
| BWS image 31.png | 0.1162 | 0.3406 | Unaffected |  |  | 1 |
| BWS image 63.png | 0.9781 |  |  | 1 |  |  |
| CdLS image 4.png | 0.9786 |  |  | 1 |  |  |
| CdLSSlide121.png | 0.0984 | 0.4770 | NS |  |  |  |
| DownSlide12.png | 0.8234 |  |  | 1 |  |  |
| DownSlide45.png | 0.7663 |  |  | 1 |  |  |
| NSSlide96.png | 0.9537 |  |  | 1 |  |  |
| NSSlide8.png | 0.7638 |  |  | 1 |  |  |
| NSSlide29.png | 0.9441 |  |  | 1 |  |  |
| NSSlide6.png | 0.5334 |  |  | 1 |  |  |
| PWSSlide13.png | 0.3098 | 0.3997 | Unaffected |  | 1 |  |
| PWSSlide87.png | 0.0031 | 0.6962 | DS |  |  |  |
| RSTS1Slide3.png | 0.9187 |  |  | 1 |  |  |
| RSTS1Slide4.png | 0.4749 |  |  | 1 |  |  |

**Image sources**

*Affected images*

22q11.2 deletion syndrome (22q11DS) (Image 100 in Figure 4 from PMID 28328118; also, Courtesy: National Human Genome Research Institute, www.genome.gov) (5), Beckwith-Wiedemann syndrome (BWS) (Figure 4 from PMID 29602885) (6), Cornelia de Lange syndrome (CdLS) (Image 72 in Figure 5 from PMID 30614194) (7), Down Syndrome (DS) (Image 34 in Figure 3 from PMID 27991738; also, Courtesy: National Human Genome Research Institute, www.genome.gov) (8), Kabuki syndrome (KS) (Figure 1 from PMID 27689058) (9), Noonan syndrome (NS) (Image A in Figure 1 from PMID 32113883) (10), Prader-Willi syndrome (PWS) (Image B in Figure 6 from PMID 18781185) (11), Rubinstein-Taybi syndrome (RSTS1) (Image 24 in Figure 3 from PMID: 32985117) (12), Williams syndrome (WS) (Image 82 in Figure 4 from PMID 29681090) (13), Wolf-Hirschhorn syndrome (WHS) (Figure 1 from PMID 30289612) (14).

*Unaffected images*

All unaffected images are from Pixabay, which allows free use according to their terms of service (see: <https://pixabay.com>). URLs for the unaffected images used in the experiments described here (as URLs may change, the descriptions in the URL links below may also be used to find these images):

[https://pixabay.com/es/photos/ni%C3%B1a-ni%C3%B1o-retrato-rosado-rubia-1018414/](https://gcc02.safelinks.protection.outlook.com/?url=https%3A%2F%2Fpixabay.com%2Fes%2Fphotos%2Fni%25C3%25B1a-ni%25C3%25B1o-retrato-rosado-rubia-1018414%2F&data=05%7C01%7Csolomonb%40mail.nih.gov%7C7c10e18bd95748f77a7d08db99ce7729%7C14b77578977342d58507251ca2dc2b06%7C0%7C0%7C638272888124683145%7CUnknown%7CTWFpbGZsb3d8eyJWIjoiMC4wLjAwMDAiLCJQIjoiV2luMzIiLCJBTiI6Ik1haWwiLCJXVCI6Mn0%3D%7C7000%7C%7C%7C&sdata=zKquZ0at%2FWHRK%2Bp4a3sLCJsKt26%2B8HcCTb%2BRPSviTWg%3D&reserved=0)

[https://pixabay.com/es/photos/ni%C3%B1os-estudiantes-africano-ni%C3%B1a-1388705/](https://gcc02.safelinks.protection.outlook.com/?url=https%3A%2F%2Fpixabay.com%2Fes%2Fphotos%2Fni%25C3%25B1os-estudiantes-africano-ni%25C3%25B1a-1388705%2F&data=05%7C01%7Csolomonb%40mail.nih.gov%7C7c10e18bd95748f77a7d08db99ce7729%7C14b77578977342d58507251ca2dc2b06%7C0%7C0%7C638272888124683145%7CUnknown%7CTWFpbGZsb3d8eyJWIjoiMC4wLjAwMDAiLCJQIjoiV2luMzIiLCJBTiI6Ik1haWwiLCJXVCI6Mn0%3D%7C7000%7C%7C%7C&sdata=81TXBQTijIOswfe%2FWqMs1fKJYX%2F3dfaeksYyMz9ESiQ%3D&reserved=0)

[https://pixabay.com/es/photos/escuela-indian-school-3760340/](https://gcc02.safelinks.protection.outlook.com/?url=https%3A%2F%2Fpixabay.com%2Fes%2Fphotos%2Fescuela-indian-school-3760340%2F&data=05%7C01%7Csolomonb%40mail.nih.gov%7C7c10e18bd95748f77a7d08db99ce7729%7C14b77578977342d58507251ca2dc2b06%7C0%7C0%7C638272888124683145%7CUnknown%7CTWFpbGZsb3d8eyJWIjoiMC4wLjAwMDAiLCJQIjoiV2luMzIiLCJBTiI6Ik1haWwiLCJXVCI6Mn0%3D%7C7000%7C%7C%7C&sdata=ndTQqc0ckwnnou9Nu%2BUKsF7mUy%2BcF8ICrR4gernMMH8%3D&reserved=0)

[https://pixabay.com/es/photos/chico-adolescente-hombre-retrato-7011010/](https://gcc02.safelinks.protection.outlook.com/?url=https%3A%2F%2Fpixabay.com%2Fes%2Fphotos%2Fchico-adolescente-hombre-retrato-7011010%2F&data=05%7C01%7Csolomonb%40mail.nih.gov%7C7c10e18bd95748f77a7d08db99ce7729%7C14b77578977342d58507251ca2dc2b06%7C0%7C0%7C638272888124683145%7CUnknown%7CTWFpbGZsb3d8eyJWIjoiMC4wLjAwMDAiLCJQIjoiV2luMzIiLCJBTiI6Ik1haWwiLCJXVCI6Mn0%3D%7C7000%7C%7C%7C&sdata=XltEb6Zt3ArGrHm1X8LIKT3iVanOZtUPtvKn9NA0Vgs%3D&reserved=0)

[https://pixabay.com/es/photos/ni%C3%B1o-mirada-ojos-chico-joven-1754050/](https://gcc02.safelinks.protection.outlook.com/?url=https%3A%2F%2Fpixabay.com%2Fes%2Fphotos%2Fni%25C3%25B1o-mirada-ojos-chico-joven-1754050%2F&data=05%7C01%7Csolomonb%40mail.nih.gov%7C7c10e18bd95748f77a7d08db99ce7729%7C14b77578977342d58507251ca2dc2b06%7C0%7C0%7C638272888124683145%7CUnknown%7CTWFpbGZsb3d8eyJWIjoiMC4wLjAwMDAiLCJQIjoiV2luMzIiLCJBTiI6Ik1haWwiLCJXVCI6Mn0%3D%7C7000%7C%7C%7C&sdata=gOG3S6wPUWCuWBkt7ukYAfaR6frDh7qqi3YaquTkTpc%3D&reserved=0)

[https://pixabay.com/photos/girl-kid-portrait-child-young-5857418/](https://gcc02.safelinks.protection.outlook.com/?url=https%3A%2F%2Fpixabay.com%2Fphotos%2Fgirl-kid-portrait-child-young-5857418%2F&data=05%7C01%7Csolomonb%40mail.nih.gov%7C7c10e18bd95748f77a7d08db99ce7729%7C14b77578977342d58507251ca2dc2b06%7C0%7C0%7C638272888124683145%7CUnknown%7CTWFpbGZsb3d8eyJWIjoiMC4wLjAwMDAiLCJQIjoiV2luMzIiLCJBTiI6Ik1haWwiLCJXVCI6Mn0%3D%7C7000%7C%7C%7C&sdata=9R%2FI%2F0VEDO%2F6IqHl5utxleZ3742fnjb6XXakVCzU8dM%3D&reserved=0)

**References**

1. Duong D, Waikel RL, Hu P, Tekendo-Ngongang C, Solomon BD. Neural network classifiers for images of genetic conditions with cutaneous manifestations. HGG Adv. 2022;3(1):100053.

2. Duong D, Hu P, Tekendo-Ngongang C, Hanchard SEL, Liu S, Solomon BD, Waikel RL. Neural Networks for Classification and Image Generation of Aging in Genetic Syndromes. Front Genet. 2022;13:864092.

3. Malechka VV, Duong D, Bordonada KD, Turriff A, Blain D, Murphy E, et al. Investigating Determinants and Evaluating Deep Learning Training Approaches for Visual Acuity in Foveal Hypoplasia. Ophthalmol Sci. 2023;3(1):100225.

4. Hsieh TC, Bar-Haim A, Moosa S, Ehmke N, Gripp KW, Pantel JT, et al. GestaltMatcher facilitates rare disease matching using facial phenotype descriptors. Nat Genet. 2022;54(3):349-57.

5. Kruszka P, Addissie YA, McGinn DE, Porras AR, Biggs E, Share M, et al. 22q11.2 deletion syndrome in diverse populations. Am J Med Genet A. 2017;173(4):879-88.

6. Pandita A, Gupta S, Gupta G, Panghal A. Beckwith-Weidemann syndrome with IC2 (KvDMR1) hypomethylation defect: a novel mutation. BMJ Case Rep. 2018;2018.

7. Dowsett L, Porras AR, Kruszka P, Davis B, Hu T, Honey E, et al. Cornelia de Lange syndrome in diverse populations. Am J Med Genet A. 2019;179(2):150-8.

8. Kruszka P, Porras AR, Sobering AK, Ikolo FA, La Qua S, Shotelersuk V, et al. Down syndrome in diverse populations. Am J Med Genet A. 2017;173(1):42-53.

9. Paik JM, Lim SY. Kabuki Syndrome with Cleft Palate. Arch Plast Surg. 2016;43(5):474-6.

10. Lutz JC, Nicot R, Schlund M, Schaefer E, Bornert F, Fioretti F, Ferri J. Dental and maxillofacial features of Noonan Syndrome: Case series of ten patients. J Craniomaxillofac Surg. 2020;48(3):242-50.

11. Cassidy SB, Driscoll DJ. Prader-Willi syndrome. Eur J Hum Genet. 2009;17(1):3-13.

12. Tekendo-Ngongang C, Owosela B, Fleischer N, Addissie YA, Malonga B, Badoe E, et al. Rubinstein-Taybi syndrome in diverse populations. Am J Med Genet A. 2020;182(12):2939-50.

13. Kruszka P, Porras AR, de Souza DH, Moresco A, Huckstadt V, Gill AD, et al. Williams-Beuren syndrome in diverse populations. Am J Med Genet A. 2018;176(5):1128-36.

14. Battaglia A, Calhoun A, Lortz A, Carey JC. Risk of hepatic neoplasms in Wolf-Hirschhorn syndrome (4p-): Four new cases and review of the literature. Am J Med Genet A. 2018;176(11):2389-94.
